# An investigation on Pythagorean fuzzy $\mathfrak {F_{p}^*}$ fraction dense space using Pythagorean fuzzy frames

**DOI:** 10.1038/s41598-025-29405-4

**Published:** 2026-01-06

**Authors:** N. B. Gnanachristy, G. K. Revathi

**Affiliations:** Department of Mathematics, School of Advanced Sciences, Vellore Institute of Technology Chennai, Vandalur-Kelambakkam Road, Chennai, 600127 Tamilnadu India

**Keywords:** Pythagorean fuzzy frames, Pythagorean fuzzy $\mathfrak {F_{p}^*}$ structure space, Pythagorean fuzzy $\mathfrak {F_{p}^*}$ fraction dense space, Engineering, Mathematics and computing

## Abstract

The concept of frame is a generalisation of the concept of category of topological space open subsets. As a result, each frame acts as an open set in this context and the Pythagorean fuzzy sets is defined as a frame. The primary goal of this research unit is to investigate the behaviour of Pythagorean fuzzy frames. Pythagorean fuzzy $\mathfrak {F_{p}^*}$ structure space is defined using Pythagorean fuzzy frames. Pythagorean fuzzy $\mathcal {G^*}$ closed sets, Pythagorean fuzzy dense set, Pythagorean fuzzy nowhere dense set, Pythagorean fuzzy somewhere dense set is established in order to investigate the Pythagorean fuzzy frames defined in Pythagorean fuzzy $\mathfrak {F_{p}^*}$ structure space. Further, Pythagorean fuzzy $\mathfrak {F_{p}^*}$ continuous function is explored in this manuscript. Separation axioms of the Pythagorean fuzzy $\mathfrak {F_{p}^*}$ structure space is established in order to comprehend the Pythagorean fuzzy frame. Additionally Pythagorean fuzzy $\mathfrak {F_{p}^*}$ fraction dense space and Pythagorean fuzzy $\mathcal {P^*}$ space is defined and explored to examine the behaviour of defined Pythagorean fuzzy frames.

## Introduction

The idea of uncertainty has been one of the most important developments in science and mathematics in the twenty-first century. The traditional perspective, which holds that uncertainty is undesirable in research and should be avoided at all costs has gradually given way to an alternate approach which is tolerant of uncertainty and holds that science cannot escape it. In an effort to address these challenges Zadeh developed the idea of fuzzy sets in 1965 to mathematically describe ambiguity. He did this by giving each member of a given set a certain grade of membership. A fuzzy set can be mathematically defined by giving each feasible individual in the universal of discourse a value that represents their degree of participation in the fuzzy set. The non-membership function was then introduced by Atanassov^1^. Pythagorean fuzzy sets were introduced by Yager^2^ as an extension of intuitionistic fuzzy sets. These Pythagorean fuzzy sets are described in this article as being framed under a few criteria. A category of open subsets in a space that may be more general than a topological space is comparable to a frame. Anything that has a collection of open subsets that function essentially like topological space open subsets can be used to define this. Currently, studies have emphasized the concept of frame category. This attempt to visualize frames makes use of the Pythagorean fuzzy set.

In this study, the novelty lies in the introduction and comprehensive examination of Pythagorean fuzzy frames as a generalization of open sets in topological spaces. Several classical topological notions such as closed sets, dense sets, nowhere dense sets, and somewhere dense sets are extended to the Pythagorean fuzzy environment within the newly developed Pythagorean fuzzy $\mathfrak {F_{p}^*}$ structure space. Moreover, the study introduces the separation axioms and investigates the characteristics of fraction dense spaces, $\mathcal {P^*}$ spaces and Pythagorean fuzzy $\mathfrak {F_{p}^*}$ continuous functions which have not been previously explored in this setting. This novel study provides a new and meaningful perspective for analyzing and generalizing topological properties within the Pythagorean fuzzy context.

## Review of literature

The term frame was introduced by Duffin and Schaeffer^3^ in non-harmonic fourier series. Later Dowker and Papert^4^ first studied frames in topology. He defined the complete lattice as the open subsets of a topological space^5^. They also demonstrated that the non-tautological statement of point-set topology can be verified in frame theory, or topology without points. Structured frames has been studied by Frith^6^. He established the category of uniform frames and quasi uniform frames. He also investigated the links between various frames. Later paracompactness is studied using frames by Pultr and Ulehla^7^. This study defined frames as paracompact and properties of paracompact frames were examined. This study also proved the frames are normal. Closure and compactness of frames were also studied by Masuret^8^. Rajesh and Thrivikraman^9^ investigated frames in fuzzy and intuitionistics fuzzy contexts. Various properties of fuzzy frames and intuitionistic frames were discussed in this study. Later Lattice valued fuzzy frames(L-Frames) were discussed by El-Saady^10^. This study defined the concept of L-fuzzy sub-frames of a given ordinary frame related to traditional frames analogously to how L-fuzzy topological spaces related to L-topological spaces. Some properties of L-fuzzy sub-frames are explored. The notion of the existence of L-fuzzy sub-frames of a particular ordinary frame was put forward in this work in the same way as L-fuzzy topological spaces were defined in relation to L-topological spaces. Also^11^ Studies in categorical topology have examined the relationship between topological spaces and frames in presheaf toposes of *M*sets, exploring internalizations, functorial connections, and conditions for adjunctions, with special focus on sobriety and spatiality when *M* is a group. Later fuzzy frames were studied via fuzzy posets Yao^12^. Yao’s intention was to define an L-frame using an L-ordered set that included more restrictions. All of these works illustrate how frames have been investigated in a variety of circumstances. Frames are explored in fraction dense space in this article. Zhang^13^ investigated a general frame in intuitionistic rough set and defined intuitionistic fuzzy relation and its properties using lattice. Thumbakara^14^defined intuitionistic fuzzy frame and coframe and also explored intuitionistic fuzzy filters in coframe context^15^. have explored ideals generated by frame homomorphisms where structures are used to form frame congruences and sublocales and the resulting locale is analyzed for compactness conditions linking algebraic and topological properties^16^, have explored semilattice-based structures such as S-bases, D-bases, and L-bases to generalize frame completeness properties and unify classical classes like zero-dimensional, completely regular, and coherent frames.. These classical study paved way to define a structure space using frames in Pythagorean fuzzy context in the present study. Furthermore, continuity is defined in the present study based on^17–21^ and these are the basic study to define the fuzzy continuous function. So these references are reviewed to define continuity in Pythagorean fuzzy frame. Separation axioms are also included based on the study of^16,22–26^. Since these separation axioms gives the detail study of open sets. The separation axioms defined in the study is based on above mentioned study. The concept of fraction dense space was given by Hager and Martinez^27^ in algebra. It was insisted that Fraction-dense algebras arise naturally in the consideration of quotient rings, and they give rise to an interesting class of topological spaces.

### Contribution of the study

The paramount goal of this scholarly article is to analyse the behaviour of Pythagorean fuzzy frames which is defined as Pythagorean fuzzy sets in various spaces.

(i) The paper constructively deal with the an introduction of Pythagorean fuzzy frames, which is novel and represents an extension of existing intuitionistic fuzzy frames.
(ii) Pythagorean fuzzy $\mathfrak {F_{p}^*}$ structure space is established using Pythagorean fuzzy frames. Then Pythagorean fuzzy $\mathcal {G^*}$ closed sets is defined. Also continuity, separation axioms of the Pythagorean fuzzy $\mathfrak {F_{p}^*}$ structure space are meticulously investigated in order to study the Pythagorean fuzzy $\mathfrak {F_{p}^*}$ open sets of the Pythagorean fuzzy $\mathfrak {F_{p}^*}$ structure space.
(iii) Pythagorean fuzzy $\mathfrak {F_{p}^*}$ structure space is carving the path toward the conceptualization of Pythagorean fuzzy $\mathfrak {F_{p}^*}$ fraction dense space.
(iv) The conceptual framework Pythagorean Fuzzy $\mathfrak {F_{p}^*}$ fraction dense space enhances the investigation of how the frames behave as sets in the new space established.
(v) The Pythagorean fuzzy $\mathcal {P^*}$ space is defined to study the relationship between the PF$\mathfrak {F_{p}^*}$RCS and Pythagorean fuzzy $\mathcal {G^*}$ closed sets.

### Structure of the paper

**Fig. 1:**
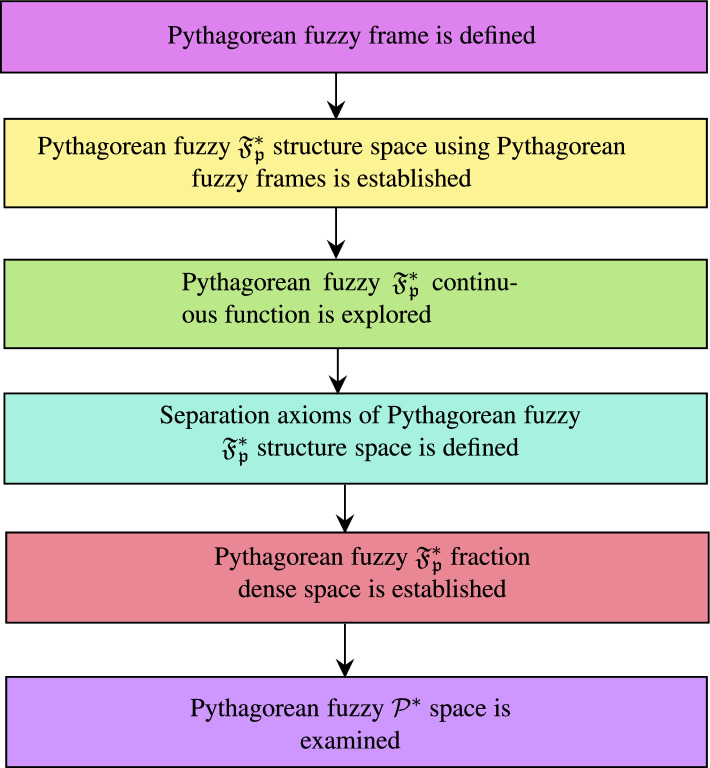
Framework of the Pythagorean fuzzy $\mathfrak {F_{p}^*}$ fraction dense space.

In this study, section “Preliminaries”, consists of the basic definitions used for the study. Section “Pythagorean fuzzy frame (PFF)”, gives the definition for Pythagorean fuzzy frames and Pythagorean fuzzy $\mathfrak {F}_{p^*}$ structure space is explained. Also Pythagorean fuzzy $\mathcal {G^*}$ closed set is defined in the Pythagorean fuzzy $\mathfrak {F}_{p^*}$ structure space. Various properties of Pythagorean fuzzy $\mathfrak {F}_{p^*}$ structure space is discussed. In section 6, Pythagorean fuzzy $\mathfrak {F}_{p^*}$ fraction dense space is defined. In section 7, Pythagorean fuzzy $\mathcal {P^*}$ space defined and the characterisations are explored. The flowchart provides the framework of the study Fig. 1.

## Preliminaries

This section provides the basic definition for this study and the nomenclature used for this study is given in the Table 1.

**Table 1:** Nomenclature of this study.

Expansion	Abbreviation
Pythagorean fuzzy frame	PFF
Pythagorean fuzzy $\mathfrak {F}_p^*$ structure space	PF$\mathfrak {F}_p^*$SS
Pythagorean fuzzy $\mathfrak {F}_p^*$ open set	PF$\mathfrak {F}_p^*$OS
Pythagorean fuzzy $\mathfrak {F}_p^*$ closed set	PF$\mathfrak {F}_p^*$CS
Pythagorean fuzzy $\mathcal {G}$ closed set	PF$\mathcal {G}$CS
Pythagorean fuzzy $\mathcal {G}^*$ closed set	PF$\mathcal {G}^*$CS
Pythagorean fuzzy $\mathfrak {F}_p^*$ regular open set	PF$\mathfrak {F}_p^*$ROS
Pythagorean fuzzy $\mathfrak {F}_p^*$ regular closed set	PF$\mathfrak {F}_p^*$RCS
Pythagorean fuzzy dense set	PFDS
Pythagorean fuzzy nowhere dense set	PFnWDS
Pythagorean somewhere dense set	PFsWDS
Pythagorean fuzzy cs-dense set	PFcsDS
Pythagorean fuzzy $\mathfrak {F}_p^*$ continuous function	PF$\mathfrak {F}_p^*$CF
Pythagorean fuzzy $\mathfrak {F}_p^*$ fraction dense space	PF$\mathfrak {F}_p^*$FDS
Pythagorean fuzzy $\mathcal {P^*}$ space	PF$\mathcal {P^*}$ S

### **Definition 1**

^28^A lattice is the partial ordered elements of the power set $\mathcal {P}(X)$ of a universal set $\mathfrak {X}$ (or any subset of $\mathfrak {X}$) can be ordered by the set inclusion S in which the join (least upper bound, supremum) and meet (greatest lower bound, infimum) of any pair of sets $A,B \in \mathcal {P}(X)$ is given by $A \cup B$ and $A\cap B$, respectively.

### **Definition 2**

^5^A partially ordered set(poset) is a set *L* with a relation $\leqq$, such that

1. if $a\leqq b$ and $b \leqq a$ then $a \leqq b$ and
2. if $a\leqq b$ and $b \leqq c$ then $a \leqq c$.

### **Definition 3**

^5^A complete lattice is a partially ordered set such that every subset *A* of *L* has a least upper bound. The least upper bound is unique and usually called the join of *A* and written as $\bigvee A$ or in terms of elements, $\bigvee a_{\alpha }$ or $a_1 \vee a_2$.

### **Definition 4**

^29^A frame is a complete lattice *L* satisfying the distributivity law $(\bigvee A)\wedge b = \bigvee \{a\wedge b | a \in A\}$ for any subset $A \subseteq L$ and any $b \in L$.

### **Definition 5**

^30^A Pythagorean fuzzy set *R* of $\mathfrak {X}\ne 0$ is a pair $(\mu _{R},\nu _{R})$ such that ${\mu _R}^2\left( x\right) +{\ \nu _R}^2\left( x\right) ={\ r_R}^2\left( x\right)$ for any $x\ \epsilon \ \mathfrak {X}$ where the fuzzy set $\mu _{R}$,$\nu _{R}$ are the membership value and non-membership value respectively and $r_R$ is the strength of commitment at a point.

### **Definition 6**

^30^Let $\tau$ be a family of Pythagorean fuzzy set of $\mathfrak {X} \ne \emptyset$. If

(i) $$0_{\mathfrak {X}},\ 1_{\mathfrak {X}}\ \epsilon \ \tau$$
(ii) ${{A_{i}}} \ {i\ \epsilon \ I} \subset \ \tau$, we have ${\bigcup \ {{{A}_i}}}_{i\ \epsilon \ I} \ A_i \ \epsilon \ \tau$ where *I* is an arbitrary index set .
(iii) $A_{1},A_{2}\ \epsilon \ \tau$, then $A_{1}\bigcup \ A_{2}\ \epsilon \ \tau$, where $0_{\mathfrak {X}}=\ (0,1)$ and $1_{\mathfrak {X}}= (1,0)$, then $\tau$ is called a Pythagorean fuzzy topology on $\mathfrak {X}$ and the pair $(\mathfrak {X},\tau )$ be a Pythagorean fuzzy topological space.

### **Definition 7**

^30^Let $S=(\mu _{S},\nu _{S})$ and $R =(\mu _{R},\nu _{R})$ be two Pythagorean fuzzy sets of a non-empty set $\mathfrak {X}$. Then,

(iv) $R \subset S$ or $S\supset R$ if $\mu _{R} \le \ \mu _{S}$ and $\nu _{R}\ge \nu _{S}.$

### **Definition 8**

^31^Let $(\mathfrak {X},\tau )$ be a Pythagorean fuzzy topological space and $R=(\mu _{R}, \nu _{R})$ be a Pythagorean fuzzy set in $\mathfrak {X}$. Then the Pythagorean fuzzy interior and Pythagorean fuzzy closure are defined by,

(i) *int*(*R*)= $\bigcup${*G*|*G* is a PFOS in $\mathfrak {X}$ and $G\subseteq R$}
(ii) *cl*(*R*)= $\bigcap${*K*|*K* is a PFCS in $\mathfrak {X}$ and$\ R\subseteq K$}

## Pythagorean fuzzy frame (PFF)

In this section PFF and Pythagorean fuzzy $\mathfrak {F}_{p^*}$ structure space(PF$\mathfrak {F}_p^*$SS) is defined using PFFs. Further Pythagorean fuzzy $\mathcal {G}^*$ closed set(PF$\mathcal {G^*}$CS) is defined in Pythagorean fuzzy $\mathfrak {F}_p^*$ structure space. Various sets Pythagorean fuzzy dense set, Pythagorean fuzzy nowhere dense sets, Pythagorean fuzzy somewhere dense set, Pythagorean fuzzy cs-dense set is also defined and the continuous function of the defined PF$\mathfrak {F}_p^*$SS is discussed.

### **Definition 9**

Let $\mathfrak {F}$ be the frame in $\mathfrak {X}$, then the Pythagorean fuzzy set $P =(\mu _{P}(x),\nu _{P}(x),x \in \mathfrak {F})$ is said to be PFF in $\mathfrak {F}$, if it satisfies the following conditions:

(i) $\mu _{P}(\vee S) \supseteq inf \{\mu _{P}(a)|a \in S \}$ $\nu _{P}(\vee S) \subseteq sup \{\nu _{P}(a)|a \in S \}$ for every arbitrary $S \subset \mathfrak {F}$.
(ii) $\mu _{P}(a \wedge b) \supseteq min \{\mu _{P}(a),\nu _{P}(a)\}$ $\nu _{P}(a \wedge b) \subseteq max \{\mu _{P}(a),\nu _{P}(a)\}$for every $a,b \in \mathfrak {F}$.
(iii) $\mu _{P}(e_{\mathfrak {F}})=\mu _{P}(O_{\mathfrak {F}})\supseteq \mu _{P}(a)$ $\nu _{P}(e_{\mathfrak {F}})= \nu _{P}(O_{\mathfrak {F}})\subseteq \nu _{P}(a)$ for all $a \in \mathfrak {F}$ where $e_{\mathfrak {F}}$ and $O_{\mathfrak {F}}$ are unit and zero element of the frame $\mathfrak {F}$.

### *Example 1*

Let $\mathfrak {F}=\{\mathfrak {X},\emptyset , \{b,c\},\{c,a\},\{a\}\}$ on $\mathfrak {X}$ where $\mathfrak {X} =\{a,b,c\}$ be the frame and $P=(\mu _{P}(x),\nu _{P}(x),x \in \mathfrak {F})$ where $\mu _{P}(\mathfrak {X})=\mu _{P}(\emptyset )=1_\mathfrak {X}, \mu _{P}(\{b,c\})=0.2, \mu _{P}(\{c,a\})=0.4, \mu _{P}(\{c\})=0.5$

$\nu _{P}(\mathfrak {X})=\nu _{P}(\emptyset )=0_\mathfrak {X}, \nu _{P}(\{b,c\})=0.7, \mu _{P}(\{c,a\})=0.7, \mu _{P}(\{c\})=0.3$ is a PFF of $\mathfrak {F}$.

### **Definition 10**

Let $\mathfrak {F}$ be the frame of any non-empty set $\mathfrak {X}$ and let $\mathfrak {F_{p}^*}$ be a collection of PFFs. If this collection satisfies the following axioms

(i) $$0_{\mathfrak {X}}$$,$$1_{\mathfrak {X}} \in \mathfrak {F_{p}^*}$$
(ii) for any $\varrho _1, \varrho _2 \in \mathfrak {F_{p}^*}$, where have $\varrho _1 \cap \varrho _2 \in \mathfrak {F_{p}^*}$
(iii) for any $\{\varrho _i\}_{i \in I} \in \mathfrak {F_{p}^{*}}$ $\cup \varrho _{i} \in \mathfrak {F_{p}^*}$, then ($\mathfrak {X}, \mathfrak {F_{p}^*}$) is called Pythagorean fuzzy $\mathfrak {F_{p}^*}$structure space(PF$\mathfrak {F_{p}^*}$SS). Each member in ($\mathfrak {X}, \mathfrak {F_{p}^*}$) is Pythagorean fuzzy $\mathfrak {F_{p}^*}$ open set(PF$\mathfrak {F_{p}^*}$OS) and its complement is called Pythagorean fuzzy $\mathfrak {F_{p}^*}$ closed set(PF$\mathfrak {F_{p}^*}$CS).

### *Example 2*

Consider the frame $\mathfrak {F}=\{\mathfrak {X}, \emptyset , \{b,c\},\{c,a\},\{a\}\}$. The PFFs $\mathcal {P},\mathcal {Q},\mathcal {R}$ are defined as $\mathcal {P}=\{(\mu _{\mathcal {P}}(x),\nu _{\mathcal {P}}(x))|x \in \mathfrak {F}\}$, $\mathcal {Q}=\{(\mu _{\mathcal {Q}}(x),\nu _{\mathcal {Q}}(x))|x \in \mathfrak {F}\}$, $\mathcal {R}=\{(\mu _{\mathcal {R}}(x),\nu _{\mathcal {R}}(x))|x \in \mathfrak {F}\}$ where,$\mu _{\mathcal {P}}(\mathfrak {X})=\mu _{\mathcal {P}}(\emptyset )=1_\mathfrak {X}, \mu _{P}(\{b,c\})=0.2, \mu _{P}(\{c,a\})=0.4, \mu _{P}(\{c\})=0.5$

$$\nu _{\mathcal {P}}(\mathfrak {X})=\nu _{\mathcal {P}}(\emptyset )=0_\mathfrak {X}, \nu _{P}(\{b,c\})=0.7, \nu _{P}(\{c,a\})=0.7, \nu _{P}(\{c\})=0.3$$

$$\mu _{\mathcal {Q}}(\mathfrak {X})=\mu _{\mathcal {Q}}(\emptyset )=1_\mathfrak {X}, \mu _{Q}(\{b,c\})=0.2, \mu _{Q}(\{c,a\})=0.5, \mu _{Q}(\{c\})=0.3$$

$$\nu _{\mathcal {Q}}(\mathfrak {X})=\nu _{\mathcal {Q}}(\emptyset )=0_\mathfrak {X}, \nu _{Q}(\{b,c\})=0.7, \nu _{Q}(\{c,a\})=0.5, \nu _{Q}(\{c\})=0.6$$

$$\mu _{\mathcal {R}}(\mathfrak {X})=\mu _{\mathcal {R}}(\emptyset )=1_\mathfrak {X}, \mu _{R}(\{b,c\})=0.6, \mu _{R}(\{c,a\})=0.4, \mu _{R}(\{c\})=0.3$$

$$\nu _{\mathcal {R}}(\mathfrak {X})=\nu _{\mathcal {R}}(\emptyset )=0_{\mathfrak {X}}, \nu _{R}(\{b,c\})=0.3, \nu _{R}(\{c,a\})=0.5, \nu _{R}(\{c\})=0.3$$.

Therefore the collection of PFFs $\mathfrak {F_{p}^*} =\{0_{\mathfrak {X}},1_{\mathfrak {X}},\mathcal {P},\mathcal {Q},\mathcal {R}\}$ is a PF$\mathfrak {F_{p}^*}$ structure. Then the structure $(\mathfrak {X}, \mathfrak {F_{p}^*})$ is a PF$\mathfrak {F_{p}^*}$SS.

### **Definition 11**

Pythagorean fuzzy $\mathfrak {F_{p}^*}$ closure and Pythagorean fuzzy $\mathfrak {F_{p}^*}$ interior of a PFS is defined by,

$cl_{\mathfrak {F_{p}^*}}(\xi ) = \cap \{\eta :\xi \le \eta ;\eta$ is $PF{\mathfrak {F_{p}^*}}$ closed in $(\mathfrak {X}, \mathfrak {F_{p}^*})\}$ $int_{\mathfrak {F_{p}^{*}}}(\xi ) = \cup \{ \sigma : \sigma \le \xi ; \sigma$ is $PF{\mathfrak {F_{p}^*}}$ open in $( \mathfrak {X}, \mathfrak {F_{p}^*} )\}$

### **Definition 12**

A PFF *P* of a PF$\mathfrak {F_{p}^*}$SS $(\mathfrak {X}, \mathfrak {F_{p}^*} )$ is called PF$\mathcal {G}$CS if $cl_{\mathfrak {F_{p}^*}}(P) \subseteq \mathcal {U}$ whenever $P \subseteq \mathcal {U}$ where $\mathcal {U}$ is a PF$\mathfrak {F_{p}^*}$OS and $\mathcal {U}\ne 0_{\mathfrak {X}} or\ 1_{\mathfrak {X}}$. The counterpart of PF$\mathcal {G}$CS is the PF$\mathcal {G}$OS.

***Notation:*** ${\mathcal {G}\ }_{\mathfrak {X}}$ will indicate the assortment of all PF$\mathcal {G}$CS in $\mathfrak {X}$.

### **Definition 13**

A PFF *P* of a PF$\mathfrak {F_{p}^*}$SS $(\mathfrak {X}, \mathfrak {F_{p}^*} )$ is called PF$\mathcal {G}$CS if $cl(P)\subseteq \mathcal {U}$ whenever$P \subseteq \mathcal {U}$ where $\mathcal {U}$ is a PFOS and $\mathcal {U}\ne 0_{X} or 1_{X}$. The counterpart of PF$\mathcal {G}$CS is the PF$\mathcal {G}$OS.

### **Definition 14**

The collection $\ {\mathcal {G}\ }_{\mathfrak {X}}\cup \{0_{\mathfrak {X}},1_{\mathfrak {X}}\}$ is PF$\mathcal {G^*}$CS in $(\mathfrak {X}, \mathfrak {F_{p}^*} )$. The counterpart of PF$\mathcal {G}^*$CS is PF$\mathcal {G}^*$OS.

***Notation:*** ${\mathcal {G}^*}_{\mathfrak {X}}$ will indicate the assortment of all PF$\mathcal {G^*}$CS in $(\mathfrak {X}, \mathfrak {F_{p}^*} )$.

### **Definition 15**

Let $( \mathfrak {X},\mathfrak {F_{p}^*})$ be PF$\mathfrak {F_{p}^*}$SS. A PFF *R* is called a Pythagorean fuzzy $\mathfrak {F_{p}^*}$ regular open set (PF$\mathfrak {F_{p}^*}$ROS) if and only if $R=int_{\mathfrak {F_{p}^*}}(cl_{\mathfrak {F_{p}^*}}(R))$; A PFS *S* is called a Pythagorean fuzzy $\mathfrak {F_{p}^*}$ regular closed (PF$\mathfrak {F_{p}^*}$RCS) if and only if $S=cl_{\mathfrak {F_{p}^*}}(int_{\mathfrak {F_{p}^*}}(S))$

### **Proposition 1**

*Let* $( \mathfrak {X},\mathfrak {F_{p}^*})$ *be PF*$\mathfrak {F_{p}^*}$*SS, Then*

(i) The closure of PF$\mathfrak {F_{p}^*}$OS is a PF$\mathfrak {F_{p}^*}$RCS.
(ii) The interior of PF$\mathfrak {F_{p}^*}$CS is a PF$\mathfrak {F_{p}^*}$ROS.

### *Proof*

(*i*) Let *K* be a PF$\mathfrak {F_{p}^*}$OS in $( \mathfrak {X}, \mathfrak {F_{p}^*})$. Clearly, $int_{\mathfrak {F_{p}^*} }(cl_{\mathfrak {F_{p}^*} }(K))\subseteq cl_{\mathfrak {F_{p}^*} }(K)$ implies that $cl_{\mathfrak {F_{p}^*} }(int_{\mathfrak {F_{p}^*}}cl_{\mathfrak {F_{p}^*} }(K)) \subseteq cl_{\mathfrak {F_{p}^*} }(K)$. Now *K* is open implies that $K \subseteq int_{\mathfrak {F_{p}^*} }(cl_{\mathfrak {F_{p}^*} }(K))$ and hence $cl_{\mathfrak {F_{p}^*} }(K)\subseteq cl_{\mathfrak {F_{p}^*} }(int_{\mathfrak {F_{p}^*} }cl_{\mathfrak {F_{p}^*} }(K))$. Thus $cl_{\mathfrak {F_{p}^*} }(K)$ is a PF$\mathfrak {F_{p}^*}$RCS.

(*ii*) The proof is similar to (*i*) . $\square$

### Proposition 2

*For a PFF* *E* *of a PF*$\mathfrak {F_{p}^*}$*SS* $( \mathfrak {X},\mathfrak {F_{p}^*})$. *Then*

(i) $$[int_{\mathfrak {F_{p}^*}}(K)]^c= cl_{\mathfrak {F_{p}^*}}(K)^c$$.
(ii) $$[cl_{\mathfrak {F_{p}^*}}(K)]^c= int_{\mathfrak {F_{p}^*}}(K)^c$$.

### *Proof*

(*i*) $$[int_{\mathfrak {F_{p}^*}}(K)]^c =[\bigcup \{L: L^c \in ( \mathfrak {X}, \mathfrak {F_{p}^*}) L \subseteq K \}]^c$$

$$= \bigcap \{L^c \in ( \mathfrak {X}, \mathfrak {F_{p}^*}) L \subseteq K \}$$

$$= \{P: P \in ( \mathfrak {X}, \mathfrak {F_{p}^*}) P \supseteq K^c\}$$

$$= cl_{\mathfrak {F_{p}^*}}(K^c)$$ where $$P= L^c$$.

(*ii*) $$[cl_{\mathfrak {F_{p}^*}}(K)]^c =[\subset \{L: L^c \in ( \mathfrak {X}, \mathfrak {F_{p}^*}) L \supseteq K \}]^c$$

$$= \bigcup \{L^c \in ( \mathfrak {X}, \mathfrak {F_{p}^*}) L \supseteq K\}$$

$$= \{P: P \in ( \mathfrak {X}, \mathfrak {F_{p}^*}) P \subseteq K^c\}$$

$$= int_{\mathfrak {F_{p}^*}}(K^c)$$ where $$P= L^c$$. $$\square$$

### **Definition 16**

A PFF *K* is a PF$\mathfrak {F_{p}^*}$SS $( \mathfrak {X}, \mathfrak {F_{p}^*})$ is called

(i) PFDS if there exists no PF$\mathfrak {F_{p}^*}$CS *G* in $( \mathfrak {X}, \mathfrak {F_{p}^*})$ such that $K \subset G \subset 1_{\mathfrak {X}}$ that is $cl_{\mathfrak {F_{p}^*}}(K)=1_{\mathfrak {X}}$ in $(\mathfrak {X}, \mathfrak {F_{p}^*})$.
(ii) PFnWDS if there exists no non-zero PF$\mathfrak {F_{p}^*}$OS *F* in $(\mathfrak {X}, \mathfrak {F_{p}^*})$ such that $F\subset cl_{ \mathfrak {F_{p}^*}}(K)$ that is $int_{ \mathfrak {F_{p}^*}}cl_{ \mathfrak {F_{p}^*}}(K)=0_{\mathfrak {X}}$ in $(\mathfrak {X}, \mathfrak {F_{p}^*})$.
(iii) PFsWDS if there exists a non-zero PF$\mathfrak {F_{p}^*}$OS *G* in $(\mathfrak {X}, \mathfrak {F_{p}^*})$ such that $G \subset cl_{\mathfrak {F_{p}^*}}(K)$ that is $int_{\mathfrak {F_{p}^*}}cl_{\mathfrak {F_{p}^*}}(K) \ne 0_{\mathfrak {X}}$ in $(\mathfrak {X}, \mathfrak {F_{p}^*})$ and $K^c$ is called a complement of PFsWDS in $(\mathfrak {X}, \mathfrak {F_{p}^*})$ and is denoted as PFcs-DS in $(\mathfrak {X}, \mathfrak {F_{p}^*})$.

### **Proposition 3**

*If* *K* *is a PFsWDS in a PF*$\mathfrak {F_{p}^*}$*FDS* $(\mathfrak {X}, \mathfrak {F_{p}^*})$, *then there exists a PF*$\mathfrak {F_{p}^*}$*RCS* *N* *in* $(\mathfrak {X}, \mathfrak {F_{p}^*})$ *such that* $N \subseteq cl_{\mathfrak {F_{p}^*}}(K)$.

### *Proof*

Let *E* be a PFsWDS in $(\mathfrak {X}, \mathfrak {F_{p}^*})$. Then, there exists PF$\mathfrak {F_{p}^*}$OS in $(\mathfrak {X}, \mathfrak {F_{p}^*})$ such that $F \subseteq cl_{\mathfrak {F_{p}^*}}(K)$. Now $cl_{\mathfrak {F_{p}^*}}(F) \subseteq cl_{\mathfrak {F_{p}^*}}(cl_{\mathfrak {F_{p}^*}}(K))$. Since *F* is a PF$\mathfrak {F_{p}^*}$OS by Proposition 6.2.51, the closure of *F* is a PF$\mathfrak {F_{p}^*}$RCS in $(\mathfrak {X}, \mathfrak {F_{p}^*})$. Let $cl_{\mathfrak {F_{p}^*}}(F)=N$. Then for the PFsWDS in *K* in $(\mathfrak {X}, \mathfrak {F_{p}^*})$ there exists a PF$\mathfrak {F_{p}^*}$RCS *N* in $(\mathfrak {X}, \mathfrak {F_{p}^*})$ such that $N \subseteq cl_{\mathfrak {F_{p}^*}}(K)$. $\square$

### **Proposition 4**

*If* *K* *is a PFcs-DS in PF*$\mathfrak {F_{p}^*}$*FDS* $(\mathfrak {X}, \mathfrak {F_{p}^*})$ *then*,

(i) $int_{\mathfrak {F_{p}^*}}(K)$ is not a PFDS in $(\mathfrak {X}, \mathfrak {F_{p}^*})$.
(ii) There exists a PF$\mathfrak {F_{p}^*}$ROS in $(\mathfrak {X}, \mathfrak {F_{p}^*})$ such that $int_{\mathfrak {F_{p}^*}}(K)\subseteq N$.

### *Proof*

(*i*) Let *K* be a PFcs-DS in $(\mathfrak {X}, \mathfrak {F_{p}^*})$. Then $K^c$ is a PFsWDS in $(\mathfrak {X}, \mathfrak {F_{p}^*})$. Thus $int_{\mathfrak {F_{p}^*}}(cl_{\mathfrak {F_{p}^*}}(K^c))$

$\ne 0_{\mathfrak {X}}$ in $(\mathfrak {X}, \mathfrak {F_{p}^*})$. This implies that $[cl_{\mathfrak {F_{p}^*}}int_{\mathfrak {F_{p}^*}}(K)]^c \ne 0_{\mathfrak {X}}$. So $cl_{\mathfrak {F_{p}^*}}int_{\mathfrak {F_{p}^*}}(K) \ne 1_{\mathfrak {X}}$. Hence $int_{\mathfrak {F_{p}^*}}(K)$ is not a PFDS in $(\mathfrak {X}, \mathfrak {F_{p}^*})$.

(*ii*) By (*i*) $int_{\mathfrak {F_{p}^*}}(K)$ is not a PFDS in $(\mathfrak {X}, \mathfrak {F_{p}^*})$. Then there exists a PF$\mathfrak {F_{p}^*}$CS *F* in $(\mathfrak {X}, \mathfrak {F_{p}^*})$ such that $int_{\mathfrak {F_{p}^*}}(K)\subset F \subset 1_{\mathfrak {X}}$. Thus $int_{\mathfrak {F_{p}^*}}int_{\mathfrak {F_{p}^*}}(K)\subset int_{\mathfrak {F_{p}^*}}(F)$. That is $int_{\mathfrak {F_{p}^*}}(K)\subset int(F)$ in $(\mathfrak {X}, \mathfrak {F_{p}^*})$. Since *F* is a PF$\mathfrak {F_{p}^*}$CS in $(\mathfrak {X}, \mathfrak {F_{p}^*})$. By Proposition 6.2.51 $int_{\mathfrak {F_{p}^*}}(F)$ is a PF$\mathfrak {F_{p}^*}$ROS in $(\mathfrak {X}, \mathfrak {F_{p}^*})$. Let $N =int_{\mathfrak {F_{p}^*}}(F)$. Then there exists a PF$\mathfrak {F_{p}^*}$ROS *N* in $(\mathfrak {X}, \mathfrak {F_{p}^*})$ such that $int_{\mathfrak {F_{p}^*}}(K) \subset N$. $\square$

### Continuous function in PF$\mathfrak {F_{p}^*}$SS

#### **Definition 17**

Let $( \mathfrak {X}, \mathfrak {F_{p}^*})$ and $( \mathfrak {Y}, \mathfrak {G_{p}^*})$ be any two PF$\mathfrak {F_{p}^*}$SS and let $\mathfrak {f}: (\mathfrak {X}, \mathfrak {F_{p}^*}) \rightarrow ( \mathfrak {Y}, \mathfrak {G_{p}^*})$ be a function. If for any $PF\mathfrak {F_{p}^*}OS, K$ of $( \mathfrak {Y}, \mathfrak {G_{p}^*})$, $\mathfrak {f}^{-1}(K)$ is a $PF\mathfrak {F_{p}^*}OS$ in $(\mathfrak {X}, \mathfrak {F_{p}^*})$, then $\mathfrak {f}$ is said to be a Pythagorean fuzzy $\mathfrak {F_{p}^*}$ continuous function (PF$\mathfrak {F_{p}^*}$CF).

#### **Proposition 5**

*Let* $(\mathfrak {X}, \mathfrak {F_{p}^*})$ *and* $(\mathfrak {Y}, \mathfrak {G_{p}^*})$ *be any two PF*$\mathfrak {F_{p}^*}$*SS and let* $\mathfrak {f}: (\mathfrak {X}, \mathfrak {F_{p}^*}) \rightarrow ( \mathfrak {Y}, \mathfrak {G_{p}^*})$ *be PF*$\mathfrak {F_{p}^*}$*CF. Then for every PFF in *$( \mathfrak {X}, \mathfrak {F_{p}^*})$, $\mathfrak {f}(cl_{\mathfrak {F_{p}^*}}(K)\subseteq cl_{\mathfrak {F_{p}^*}}(\mathfrak {f}(K))$.

#### *Proof*

Let *K* be a PFF in $(\mathfrak {X}, \mathfrak {F_{p}^*})$. Since $cl_{\mathfrak {F_{p}^*}}(\mathfrak {f}(K))$ is a PF$\mathfrak {F_{p}^*}$CS and $\mathfrak {f}$ is a PF$\mathfrak {F_{p}^*}$CF. $\mathfrak {f}^{-1}(cl(\mathfrak {f}(K)))$ is a PF$\mathfrak {F_{p}^*}$CS and $\mathfrak {f}^{-1}cl_{\mathfrak {F_{p}^*}}(\mathfrak {f}(K)) \supseteq K$. Now $cl_{\mathfrak {F_{p}^*}}(\mathfrak {f}(K))\subseteq \mathfrak {f}^{-1}(cl_{\mathfrak {F_{p}^*}}(\mathfrak {f}(K)))$. Therefore, $\mathfrak {f}(cl_{\mathfrak {F_{p}^*}}(K))\subseteq cl_{\mathfrak {F_{p}^*}}(\mathfrak {f}(K))$. $\square$

#### **Proposition 6**

*Let* $(\mathfrak {X}, \mathfrak {F_{p}^*})$ *and* $(\mathfrak {Y}, \mathfrak {G_{p}^*})$ *be any two PF*$\mathfrak {F_{p}^*}$*SS. If* *E* *is PF*$\mathfrak {{F_{p}^*}}$*CS in *$(\mathfrak {X}, \mathfrak {F_{p}^*})$ *and if* $\mathfrak {f}:(\mathfrak {X}, \mathfrak {F_{p}^*})\rightarrow (\mathfrak {Y}, \mathfrak {G_{p}^*})$ *be a PF*$\mathfrak {F_{p}^*}$*CF then* $\mathfrak {f}(K)$ *is a PF*$\mathfrak {F_{p}^*}$*CS in* $(\mathfrak {Y}, \mathfrak {G_{p}^*})$.

#### *Proof*

Let *K* be a PF$\mathfrak {{F_{p}^*}}$OS in $(\mathfrak {Y}, \mathfrak {G_{p}^*})$. If $\mathfrak {f}(E)\subseteq K$ then $E \subseteq \mathfrak {f}^{-1}(K)$ in $(\mathfrak {X}, \mathfrak {F_{p}^*})$. Since *E* is a PF$\mathfrak {{F_{p}^*}}$CS and $\mathfrak {f}^{-1}(K)$ is a PF$\mathfrak {{F_{p}^*}}$OS in $(\mathfrak {X}, \mathfrak {F_{p}^*})$. Then $cl_{\mathfrak {F_{p}^*}}(E)\subseteq \mathfrak {f}^{-1}(K)$ implies $\mathfrak {f}(cl_{\mathfrak {F_{p}^*}}(E)) \subseteq K$. By assumption, $\mathfrak {f}(cl_{\mathfrak {F_{p}^*}}(E))$ is PF$\mathfrak {{F_{p}^*}}$CS in $(\mathfrak {Y}, \mathfrak {G_{p}^*})$ and $cl_{\mathfrak {F_{p}^*}}(\mathfrak {f}(E))= cl_{\mathfrak {F_{p}^*}}(\mathfrak {f}(cl_{\mathfrak {F_{p}^*}}(E)))\subseteq K$. Hence $\mathfrak {f}(E)$ is PF$\mathfrak {F_{p}^*}$CS. $\square$

#### **Proposition 7**

*Let* $(\mathfrak {X}, \mathfrak {F_{p}^*})$ *and* $(\mathfrak {Y}, \mathfrak {G_{p}^*})$ *be two PF*$\mathfrak {F_{p}^*}$*SS and* $K \subseteq \mathfrak {F_{p}^*}, E \subseteq \mathfrak {G_{p}^*}$. *Then the following statements are equivalent: *

(i) $\mathfrak {f}: (\mathfrak {X}, \mathfrak {F_{p}^*}) \rightarrow (\mathfrak {Y}, \mathfrak {G_{p}^*})$ is PF$\mathfrak {F_{p}^*}$CF.
(ii) $\mathfrak {f}^{-1}(int_{\mathfrak {F_{p}^*}}(E)) \subseteq int_{\mathfrak {F_{p}^*}}(\mathfrak {f}^{-1}(E))$ for each *E* in $(\mathfrak {Y}, \mathfrak {G_{p}^*})$.
(iii) $cl_{\mathfrak {F_{p}^*}}(\mathfrak {f}^{-1}(E)) \subseteq \mathfrak {f}^{-1}(cl_{\mathfrak {F_{p}^*}}(E))$ for each *E* in $(\mathfrak {Y}, \mathfrak {G_{p}^*})$.

#### *Proof*

$(i) \Rightarrow (ii)$ from (*i*) $\mathfrak {f}: (\mathfrak {X}, \mathfrak {F_{p}^*}) \rightarrow (\mathfrak {Y}, \mathfrak {G_{p}^*})$ is PF$\mathfrak {F_{p}^*}$CF. Let *E* be a Pythagorean fuzzy frame. By the definition of PF$\mathfrak {F_{p}^*}$CF $\mathfrak {f}^{-1}(int_{\mathfrak {F_{p}^*}}(E))$ is a PFF in $(\mathfrak {X}, \mathfrak {F_{p}^*})$. $\mathfrak {f}^{-1}(E)$ is a PFF in $(\mathfrak {X}, \mathfrak {F_{p}^*})$ then $int_{\mathfrak {F_{p}^*}}(\mathfrak {f}^{-1}(E))$ is a PFF in $(\mathfrak {X}, \mathfrak {F_{p}^*})$. Therefore, $\mathfrak {f}^{-1}(int_{\mathfrak {F_{p}^*}}(E)) \subseteq int_{\mathfrak {F_{p}^*}}(\mathfrak {f}^{-1}(E))$.

$(ii) \Rightarrow (iii)$. Given $\mathfrak {f}^{-1}(int_{\mathfrak {F_{p}^*}}(E)) \subseteq int_{\mathfrak {F_{p}^*}}(\mathfrak {f}^{-1}(E))$. Let *E* be a PFF in $(\mathfrak {X}, \mathfrak {F_{p}^*})$. Let *E* be a PF$\mathfrak {F_{p}^*}$OS in $(\mathfrak {Y}, \mathfrak {G_{p}^*})$. Since $int_{\mathfrak {F_{p}^*}}(E)=E$, $int_{\mathfrak {F_{p}^*}}(\mathfrak {f}^{-1}(E))=\mathfrak {f}^{-1}(E)$. By (*i*) $\mathfrak {f}^{-1}(int_{\mathfrak {F_{p}^*}}(E))=int_{\mathfrak {F_{p}^*}}(\mathfrak {f}^{-1}) (E)$. Therefore $\mathfrak {f}^{-1} (E) \subseteq int_{\mathfrak {F_{p}^*}}(\mathfrak {f}^{-1} (E))$. Hence $\mathfrak {f}^{-1} (E)= int_{\mathfrak {F_{p}^*}}\mathfrak {f}^{-1} (E)$. Therefore $\mathfrak {f}^{-1} (E)$ is a PFF in $(\mathfrak {X}, \mathfrak {F_{p}^*})$. Hence $\mathfrak {f}$ is PF$\mathfrak {F_{p}^*}$CF.

$(i) \Rightarrow (iii)$ Given $\mathfrak {f}: (\mathfrak {X}, \mathfrak {F_{p}^*}) \rightarrow (\mathfrak {Y}, \mathfrak {G_{p}^*})$ is PF$\mathfrak {F_{p}^*}$CF. Let *E* is a PFF in $(\mathfrak {Y}, \mathfrak {G_{p}^*})$ and $E= \mathfrak {f}^{-1}(K) \Rightarrow \mathfrak {f}(E)=\mathfrak {f}(\mathfrak {f}^{-1}(K))\subseteq K$. By Proposition 6.2.55, $\mathfrak {f}(cl_{\mathfrak {F_{p}^*}}\mathfrak {f}^{-1}(K)) \subseteq cl_{\mathfrak {F_{p}^*}}(\mathfrak {f}\mathfrak {f}^{-1}(K))$. Thus $cl_{\mathfrak {F_{p}^*}}\mathfrak {f}^{-1}(K) \subseteq \mathfrak {f}^{-1}cl_{\mathfrak {F_{p}^*}}(K)$.

$(iii) \Rightarrow (i)$ $\mathfrak {f}^{-1}int_{\mathfrak {F_{p}^*}}(E) \subseteq int_{\mathfrak {F_{p}^*}}\mathfrak {f}^{-1}(E)$. To prove $\mathfrak {f}: (\mathfrak {X}, \mathfrak {F_{p}^*}) \rightarrow (\mathfrak {Y}, \mathfrak {G_{p}^*})$ is PF$\mathfrak {F_{p}^*}$CF. It is enough to prove the inverse image of each PFF in $(\mathfrak {Y}, \mathfrak {G_{p}^*})$ is a PFF in $(\mathfrak {X}, \mathfrak {F_{p}^*})$. Let *E* be a PFF in $(\mathfrak {Y}, \mathfrak {G_{p}^*})$. To show that $\mathfrak {f}^{-1}(E)$ is PFF in $(\mathfrak {X}, \mathfrak {F_{p}^*})$. Since $E=cl_{\mathfrak {F_{p}^*}}(E)$. $\mathfrak {f}^{-1}(E)=\mathfrak {f}^{-1}cl_{\mathfrak {F_{p}^*}}(E)$ but $cl_{\mathfrak {F_{p}^*}}\mathfrak {f}^{-1}(E) \subseteq \mathfrak {f}^{-1}cl_{\mathfrak {F_{p}^*}}(E)$. Hence $cl_{\mathfrak {F_{p}^*}}\mathfrak {f}^{-1}(E) \subseteq \mathfrak {f}^{-1}(E)= \mathfrak {f}^{-1}cl_{\mathfrak {F_{p}^*}}(E)$. Therefore $\mathfrak {f}^{-1}(E)=cl_{\mathfrak {F_{p}^*}}\mathfrak {f}^{-1}(E) \Rightarrow$ is PFF in $(\mathfrak {X}, \mathfrak {F_{p}^*})$. This proves $\mathfrak {f}$ is a PF$\mathfrak {F_{p}^*}$CF. $\square$

## Separation axioms on Pythagorean fuzzy $\mathfrak {F_{p}^*}$ structure space

In this section, separation axioms are discussed on Pythagorean fuzzy $\mathfrak {F_{p}^*}$ structure space in detail. Four different $T_0,T_1,T_2$ spaces are defined and the characterisations are investigated.

### **Definition 18**

A $$PF\mathfrak {F_{p}^*}SS$$ $$(\mathfrak {X},\mathfrak {F_{p}^*})$$ is called

(i) $\mathfrak {F_{p}^*} T_0$ space a) if for all $x,y \in \mathfrak {X}, x \ne y$ there exists a $PF\mathfrak {F_{p}^*}OS, U=(\mu _{U},\nu _{U}),V=(\mu _{V},\nu _{V}) \in \mathfrak {F_{p}^*}$ such that $\mu _{U}(x)=1,\nu _{U}(x)=0,\mu _{U}(y)=0,\nu _{U}(y)=1$ or $\mu _{V}(x)=1,\nu _{V}(x)=0,\mu _{V}(y)=0,\nu _{V}(y)=1$.
(ii) $\mathfrak {F_{p}^*} T_0$ space b) if for all $x,y \in \mathfrak {X}, x \ne y$ there exists a $PF\mathfrak {F_{p}^*}OS, U=(\mu _{U},\nu _{U}),V=(\mu _{V},\nu _{V}) \in \mathfrak {F_{p}^*}$ such that $\mu _{U}(x)=1,\nu _{U}(x)=0,\mu _{U}(y)=0,\nu _{U}(y)>0$ or $\mu _{V}(x)=1,\nu _{V}(x)=0,\mu _{V}(y)=0,\nu _{V}(y)>0$.
(iii) $\mathfrak {F_{p}^*} T_0$ space c) if for all $x,y \in \mathfrak {X}, x \ne y$ there exists a $PF\mathfrak {F_{p}^*}OS, U=(\mu _{U},\nu _{U}),V=(\mu _{V},\nu _{V}) \in \mathfrak {F_{p}^*}$ such that $\mu _{U}(x)>0,\nu _{U}(x)=0,\mu _{U}(y)=0,\nu _{U}(y)=1$ or $\mu _{V}(x)>0,\nu _{V}(x)=0,\mu _{V}(y)=0,\nu _{V}(y)=1$.
(iv) $\mathfrak {F_{p}^*} T_0$ space d) if for all $x,y \in \mathfrak {X}, x \ne y$ there exists a $PF\mathfrak {F_{p}^*}OS, U=(\mu _{U},\nu _{U}),V=(\mu _{V},\nu _{V}) \in \mathfrak {F_{p}^*}$ such that $\mu _{U}(x)>0,\nu _{U}(x)=0,\mu _{U}(y)=0,\nu _{U}(y)>0$ or $\mu _{V}(x)>0,\nu _{V}(x)=0,\mu _{V}(y)=0,\nu _{V}(y)>0$.

**Fig. 2:**
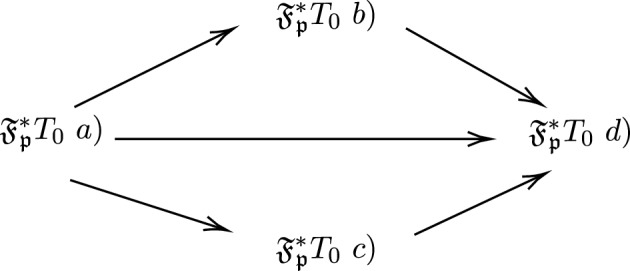
This diagram depicts the implication of $\mathfrak {F_{p}^*} T_0$ space.

### **Proposition 8**

*Let* $(\mathfrak {X},\mathfrak {F_{p}^*})$ *be a* $PF\mathfrak {F_{p}^*}SS$.* Then the following implications hold and it is given in Fig. *2

### *Proof*

To prove $\mathfrak {F_{p}^*} T_{0}\ a) \Rightarrow \mathfrak {F_{p}^*} T_{0}\ b)$. Let $(\mathfrak {X},\mathfrak {F_{p}^*})$ be a $\mathfrak {F_{p}^*} T_{0} \ a)$ by definition of $\mathfrak {F_{p}^*} T_{0} \ a)$ for all $x,y \in \mathfrak {X}, x \ne y$ there exists $U=(\mu _{U},\nu _{U}) \in \mathfrak {F_{p}^*}$ such that $\mu _{U}(x)=1, \nu _{U}(x)=0, \mu _{U}(y)=0, \mu _{U}(y)=1,\mu _{U}(z)=1,\nu _{U}(z)=0$ implies $\mu _{U}(x)=0, \nu _{U}(x)=0, \mu _{U}(y)=0, \mu _{U}(y)>0, \mu _{U}(z)=1,\nu _{U}(z)=0$, which is $\mathfrak {F_{p}^*} T_{0}\ b)$. Hence $\mathfrak {F_{p}^*} T_{0}\ a) \Rightarrow \mathfrak {F_{p}^*} T_{0}\ b)$.

Similarly, $$\mathfrak {F_{p}^*} T_{0}\ a) \Rightarrow \mathfrak {F_{p}^*} T_{0}\ c)$$

$$\mathfrak {F_{p}^*} T_{0}\ a) \Rightarrow \mathfrak {F_{p}^*} T_{0}\ d)$$,

$$\mathfrak {F_{p}^*} T_{0}\ b) \Rightarrow \mathfrak {F_{p}^*}T_{0}\ c)$$,

$$\mathfrak {F_{p}^*} T_{0}\ c) \Rightarrow \mathfrak {F_{p}^*} T_{0}\ d)$$. $$\square$$

### *Remark 1*

The converse of the above implications is not true. It can be seen through the following Examples 6.3.31, 6.3.32, 6.3.33.

### *Example 3*

Consider the frame $\mathfrak {F}=\{\mathfrak {X}, \emptyset , \{b,c\},\{c,a\},\{a\}\}$. The PFFs $\mathcal {P},\mathcal {Q},\mathcal {R}$ are defined as $\mathcal {P}=\{(\mu _{\mathcal {P}}(x),\nu _{\mathcal {P}}(x))|x \in \mathfrak {F}\}$, $\mathcal {Q}=\{(\mu _{\mathcal {Q}}(x),\nu _{\mathcal {Q}}(x))|x \in \mathfrak {F}\}$, $\mathcal {R}=\{(\mu _{\mathcal {R}}(x),\nu _{\mathcal {R}}(x))$

$$|x \in \mathfrak {F}\}$$, $$\mathcal {S}=\{(\mu _{\mathcal {S}}(x),\nu _{\mathcal {S}}(x))|x \in \mathfrak {F}\}$$ where,

$$F_{\mathcal {P}}(\mathfrak {X})=\mu _{\mathcal {P}}(\emptyset )=1_\mathfrak {X}, \mu _{P}(\{b,c\})=0.6, \mu _{P}(\{c,a\})=0.4, \mu _{P}(\{c\})=0.3$$

$$\nu _{\mathcal {P}}(\mathfrak {X})=\nu _{\mathcal {P}}(\emptyset )=0_\mathfrak {X}, \nu _{P}(\{b,c\})=0.3, \nu _{P}(\{c,a\})=0.5, \nu _{P}(\{c\})=0.3$$

$$F_{\mathcal {Q}}(\mathfrak {X})=\mu _{\mathcal {Q}}(\emptyset )=1_\mathfrak {X}, \mu _{Q}(\{b,c\})=1, \mu _{Q}(\{c,a\})=0, \mu _{Q}(\{c\})=1$$

$$\nu _{\mathcal {Q}}(\mathfrak {X})=\nu _{\mathcal {Q}}(\emptyset )=0_\mathfrak {X}, \nu _{Q}(\{b,c\})=0, \nu _{Q}(\{c,a\})=0.3, \nu _{Q}(\{c\})=0$$

$$\mu _{\mathcal {R}}(\mathfrak {X})=\mu _{\mathcal {R}}(\emptyset )=1_\mathfrak {X}, \mu _{R}(\{b,c\})=1, \mu _{R}(\{c,a\})=0.4, \mu _{R}(\{c\})=1$$

$$\nu _{\mathcal {R}}(\mathfrak {X})=\nu _{\mathcal {R}}(\emptyset )=0_{\mathfrak {X}}, \nu _{R}(\{b,c\})=0, \nu _{R}(\{c,a\})=0.3, \nu _{R}(\{c\})=0$$.

$$\mu _{\mathcal {S}}(\mathfrak {X})=\mu _{\mathcal {S}}(\emptyset )=1_\mathfrak {X}, \mu _{S}(\{b,c\})=0.6, \mu _{S}(\{c,a\})=0, \mu _{S}(\{c\})=0.3$$

$$\nu _{\mathcal {S}}(\mathfrak {X})=\nu _{\mathcal {S}}(\emptyset )=0_{\mathfrak {X}}, \nu _{S}(\{b,c\})=0.3, \nu _{S}(\{c,a\})=0.3, \nu _{S}(\{c\})=0.3$$.

Then $(\mathfrak {X}, \mathfrak {F_{p}^*})$ is a $\mathfrak {F_{p}^*} T_{0}b)$ but not $\mathfrak {F_{p}^*} T_{0}a)$.

### *Example 4*

Consider the frame $\mathfrak {F}=\{\mathfrak {X}, \emptyset , \{b,c\},\{c,a\},\{a\}\}$. The PFFs $\mathcal {P},\mathcal {Q},\mathcal {R}$ are defined as $\mathcal {P}=\{(\mu _{\mathcal {P}}(x),\nu _{\mathcal {P}}(x))|x \in \mathfrak {F}\}$, $\mathcal {Q}=\{(\mu _{\mathcal {Q}}(x),\nu _{\mathcal {Q}}(x))|x \in \mathfrak {F}\}$ where,

$$\mu _{\mathcal {P}}(\mathfrak {X})=\mu _{\mathcal {P}}(\emptyset )=1_\mathfrak {X}, \mu _{P}(\{b,c\})=0.6, \mu _{P}(\{c,a\})=0.4, \mu _{P}(\{c\})=0.3$$

$$\nu _{\mathcal {P}}(\mathfrak {X})=\nu _{\mathcal {P}}(\emptyset )=0_\mathfrak {X}, \nu _{P}(\{b,c\})=0.3, \nu _{P}(\{c,a\})=0.5, \nu _{P}(\{c\})=0.3$$

$$\mu _{\mathcal {Q}}(\mathfrak {X})=\mu _{\mathcal {Q}}(\emptyset )=1_\mathfrak {X}, \mu _{Q}(\{b,c\})=0.3, \mu _{Q}(\{c,a\})=0, \mu _{Q}(\{c\})=0.3$$

$$\nu _{\mathcal {Q}}(\mathfrak {X})=\nu _{\mathcal {Q}}(\emptyset )=0_\mathfrak {X}, \nu _{Q}(\{b,c\})=0, \nu _{Q}(\{c,a\})=1, \nu _{Q}(\{c\})=0$$

Then $(\mathfrak {X}, \mathfrak {F_{p}^*})$ is a $\mathfrak {F_{p}^*} T_{0}c)$ but not $\mathfrak {F_{p}^*} T_{0}a)$.

### *Example 5*

Consider the frame $\mathfrak {F}=\{\mathfrak {X}, \emptyset , \{b,c\},\{c,a\},\{a\}\}$. The PFFs $\mathcal {P},\mathcal {Q},\mathcal {R}$ are defined as $\mathcal {P}=\{(\mu _{\mathcal {P}}(x),\nu _{\mathcal {P}}(x))|x \in \mathfrak {F}\}$, $\mathcal {Q}=\{(\mu _{\mathcal {Q}}(x),\nu _{\mathcal {Q}}(x))|x \in \mathfrak {F}\}$ where,

$$F_{\mathcal {P}}(\mathfrak {X})=\mu _{\mathcal {P}}(\emptyset )=1_\mathfrak {X}, \mu _{P}(\{b,c\})=0.6, \mu _{P}(\{c,a\})=0.4, \mu _{P}(\{c\})=0.3$$

$$\nu _{\mathcal {P}}(\mathfrak {X})=\nu _{\mathcal {P}}(\emptyset )=0_\mathfrak {X}, \nu _{P}(\{b,c\})=0.3, \nu _{P}(\{c,a\})=0.5, \nu _{P}(\{c\})=0.3$$

$$\mu _{\mathcal {Q}}(\mathfrak {X})=\mu _{\mathcal {Q}}(\emptyset )=1_\mathfrak {X}, \mu _{Q}(\{b,c\})=0.3, \mu _{Q}(\{c,a\})=0, \mu _{Q}(\{c\})=0.3$$

$$\nu _{\mathcal {Q}}(\mathfrak {X})=\nu _{\mathcal {Q}}(\emptyset )=0_\mathfrak {X}, \nu _{Q}(\{b,c\})=0.3, \nu _{Q}(\{c,a\})=0.3, \nu _{Q}(\{c\})=0$$

Then $(\mathfrak {X}, \mathfrak {F_{p}^*})$ is a $\mathfrak {F_{p}^*} T_{0}d)$ but not $\mathfrak {F_{p}^*} T_{0}a),\mathfrak {F_{p}^*} T_{0}b,\mathfrak {F_{p}^*} T_{0}c)$.

### **Proposition 9**

*Let* $(\mathfrak {X},\mathfrak {F_{p}^*})$ *be a *$PF\mathfrak {F_{p}^*}SS$, $Q \subseteq X$ *and* $\mathcal {X}_{Q}$ *be the characteristic function of Q* *and* $\mathfrak {F_{p}^*}(Q)=\{\varrho \cap \mathcal {X}_{Q}, \varrho \in \mathfrak {F_{p}^*}\}$ *then*

(i) $$(\mathfrak {X},\mathfrak {F_{p}^*})$$ is $$\mathfrak {F_{p}^*}T_{0}a) \Rightarrow Q$$ is $$\mathfrak {F_{p}^*}T_{0}a)$$
(ii) $(\mathfrak {X},\mathfrak {F_{p}^*})$ is $\mathfrak {F_{p}^*}T_{0}b) \Rightarrow Q$ is $\mathfrak {F_{p}^*}T_{0}b)$
(iii) $(\mathfrak {X},\mathfrak {F_{p}^*})$ is $\mathfrak {F_{p}^*}T_{0}c) \Rightarrow Q$ is $\mathfrak {F_{p}^*}T_{0}c)$
(iv) $(\mathfrak {X},\mathfrak {F_{p}^*})$ is $\mathfrak {F_{p}^*}T_{0}d) \Rightarrow Q$ is $\mathfrak {F_{p}^*}T_{0}d)$

### *Proof*

Let $(\mathfrak {X},\mathfrak {F_{p}^*})$ is a $\mathfrak {F_{p}^*}T_{0}$ space a). Let $\mathfrak {F_{p}^*}(Q)=\{\varrho \cap \mathcal {X}_{Q}, \varrho \in \mathfrak {F_{p}^*}\}$. Let $x,y \in Q \subseteq \mathfrak {X}, x \ne y$ then $x,y \in \mathfrak {X}$ as $Q \subseteq \mathfrak {X}$. Since $(X,\mathfrak {F_{p}^*})$ is $\mathfrak {F_{p}^*}T_{0}$a)space then there exists $U=(\mu _{U},\nu _{U}), V=(\mu _{V},\nu _{V}) \in \mathfrak {F_{p}^*}$ such that $\mu _{U}(x)=1, \nu _{U}(x)=0, \mu _{U}(y)=0, \mu _{U}(y)=1$ and Then *R* are $PF\mathfrak {F_{p}^*}OS$ in *Q* such that $\mu _{R}(x)=1, \nu _{R}(x)=0, \mu _{R}(y)=0, \mu _{R}(y)=1$. Then *Q* is also $\mathfrak {F_{p}^*}T_{0}$a) space.

The proof of (*ii*), (*iii*), (*iv*) is obvious. $\square$

### **Definition 19**

A $$PF\mathfrak {F_{p}^*}SS$$ $$(\mathfrak {X},\mathfrak {F_{p}^*})$$ is called

(i) $\mathfrak {F_{p}^*} T_1$ space a) if for all $x,y \in \mathfrak {X}, x \ne y$ there exists a $PF\mathfrak {F_{p}^*}OS, U=(\mu _{U},\nu _{U}),V=(\mu _{V},\nu _{V}) \in \mathfrak {F_{p}^*}$ such that $\mu _{U}(x)=1,\nu _{U}(x)=0,\mu _{U}(y)=0,\nu _{U}(y)=1$ and $\mu _{V}(y)=1,\nu _{V}(y)=0,\mu _{V}(x)=0,\nu _{V}(x)=1.$
(ii) $\mathfrak {F_{p}^*} T_1$ space b) if for all $x,y \in \mathfrak {X}, x \ne y$ there exists a $PF\mathfrak {F_{p}^*}OS, U=(\mu _{U},\nu _{U}),V=(\mu _{V},\nu _{V}) \in \mathfrak {F_{p}^*}$ such that $\mu _{U}(x)=1,\nu _{U}(x)=0,\mu _{U}(y)=0,\nu _{U}(y)>0$ and $\mu _{V}(y)=1,\nu _{V}(y)=0,\mu _{V}(x)=0,\nu _{V}(x)>0.$
(iii) $\mathfrak {F_{p}^*} T_1$ space c) if for all $x,y \in \mathfrak {X}, x \ne y$ there exists a $PF\mathfrak {F_{p}^*}OS, U=(\mu _{U},\nu _{U}),V=(\mu _{V},\nu _{V}) \in \mathfrak {F_{p}^*}$ such that $\mu _{U}(x)>0,\nu _{U}(x)=0,\mu _{U}(y)=0,\nu _{U}(y)=1$ and $\mu _{V}(y)>0,\nu _{V}(y)=0,\mu _{V}(x)=0,\nu _{V}(x)=1.$
(iv) $\mathfrak {F_{p}^*} T_1$ space d) if for all $x,y \in \mathfrak {X}, x \ne y$ there exists a $PF\mathfrak {F_{p}^*}OS, U=(\mu _{U},\nu _{U}),V=(\mu _{V},\nu _{V}) \in \mathfrak {F_{p}^*}$ such that $\mu _{U}(x)>0,\nu _{U}(x)=0,\mu _{U}(y)=0,\nu _{U}(y)>0$ and $\mu _{V}(y)>0,\nu _{V}(y)=0,\mu _{V}(x)=0,\nu _{V}(x)>0.$

**Fig. 3:**
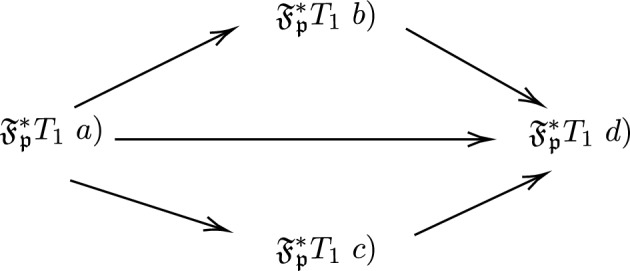
This diagram depicts the implication of $\mathfrak {F_{p}^*} T_1$ space.

### Proposition 10

*Let* $(\mathfrak {X},\mathfrak {F_{p}^*})$ *be a *$PF\mathfrak {F_{p}^*}SS$. *Then the following implications hold and it is given in Fig.* 3

### *Proof*

To prove $\mathfrak {F_{p}^*} T_{1}\ a) \Rightarrow \mathfrak {F_{p}^*} T_{1}\ b)$. Let $(\mathfrak {X},\mathfrak {F_{p}^*})$ be a $\mathfrak {F_{p}^*} T_{0} \ a)$ by definition of $\mathfrak {F_{p}^*} T_{1} \ a)$ for all $x,y \in \mathfrak {X} x \ne y$ there exists $U=(\mu _{U},\nu _{U}),V=(\mu _{V},\nu _{V}) \in \mathfrak {F_{p}^*}$ such that $\mu _{U}(x)=1, \nu _{U}(x)=0, \mu _{U}(y)=0, \mu _{U}(y)=1$ and $\mu _{U}(y)=1, \nu _{U}(y)=0, \mu _{U}(x)=0, \mu _{U}(x)=1$ implies $\mu _{U}(x)=1, \nu _{U}(x)=0, \mu _{U}(y)=0, \mu _{U}(y)>0$ $\mu _{U}(y)=1, \nu _{U}(y)=0, \mu _{U}(x)=0, \mu _{U}(x)>0$, which is $\mathfrak {F_{p}^*} T_{1}\ b)$. Hence $\mathfrak {F_{p}^*} T_{1}\ a) \Rightarrow \mathfrak {F_{p}^*} T_{1}\ b)$. Similarly, $\mathfrak {F_{p}^*} T_{1}\ a) \Rightarrow \mathfrak {F_{p}^*} T_{1}\ c)$

$$\mathfrak {F_{p}^*} T_{1}\ a) \Rightarrow \mathfrak {F_{p}^*} T_{1}\ d)$$,

$$\mathfrak {F_{p}^*} T_{1}\ b) \Rightarrow \mathfrak {F_{p}^*} T_{1}\ c)$$,

$$\mathfrak {F_{p}^*} T_{1}\ c) \Rightarrow \mathfrak {F_{p}^*} T_{1}\ d)$$. $$\square$$

### *Remark 2*

None of the above implications are true. It can be proved by the following similar examples.

### **Proposition 11**

*Let* $(\mathfrak {X},\mathfrak {F_{p}^*})$ *be a* $PF\mathfrak {F_{p}^*}SS$, $Q \subseteq X$ *and* $\mathcal {X}_{Q}$ *be the characteristic function of Q and* $\mathfrak {F_{p}^*}(Q)=\{\varrho \cap \mathcal {X}_{Q}, \varrho \in \mathfrak {F_{p}^*}\}$ *then*

(i) $$(\mathfrak {X},\mathfrak {F_{p}^*})$$ is $$\mathfrak {F_{p}^*}T_{1}a) \Rightarrow Q$$ is $$\mathfrak {F_{p}^*}T_{1}a)$$
(ii) $(\mathfrak {X},\mathfrak {F_{p}^*})$ is $\mathfrak {F_{p}^*}T_{1}b) \Rightarrow Q$ is $\mathfrak {F_{p}^*}T_{1}b)$
(iii) $(\mathfrak {X},\mathfrak {F_{p}^*})$ is $\mathfrak {F_{p}^*}T_{1}c) \Rightarrow Q$ is $\mathfrak {F_{p}^*}T_{1}c)$
(iv) $(\mathfrak {X},\mathfrak {F_{p}^*})$ is $\mathfrak {F_{p}^*}T_{1}d) \Rightarrow Q$ is $\mathfrak {F_{p}^*}T_{1}d)$

### *Proof*

Let $(\mathfrak {X},\mathfrak {F_{p}^*})$ is a $\mathfrak {F_{p}^*}T_{1}$ space a). Let $\mathfrak {F_{p}^*}(Q)=\{\varrho \cap \mathcal {X}_{Q}, \varrho \in \mathfrak {F_{p}^*}\}$. Let $x,y \in Q \subseteq \mathfrak {X}, x \ne y$ then $x,y \in \mathfrak {X}$ as $Q \subseteq \mathfrak {X}$. Since $(X,\mathfrak {F_{p}^*})$ is $\mathfrak {F_{p}^*}T_{1}$a)space then there exists $U=(\mu _{U},\nu _{U}), V=(\mu _{V},\nu _{V}) \in \mathfrak {F_{p}^*}$ such that $\mu _{U}(x)=1, \nu _{U}(x)=0, \mu _{U}(y)=0, \mu _{U}(y)=1$ and $\mu _{U}(y)=1, \nu _{U}(y)=0, \mu _{U}(x)=0, \mu _{U}(x)=1$. Then *R*, *S* are $PF\mathfrak {F_{p}^*}OS$ in *Q* such that $\mu _{R}(x)=1, \nu _{R}(x)=0, \mu _{R}(y)=0, \mu _{R}(y)=1$ and $\mu _{S}(y)=1, \nu _{S}(y)=0, \mu _{S}(x)=0, \mu _{S}(x)=1$. Then *Q* is also $\mathfrak {F_{p}^*}T_{1}$a) space.

The proof of (*ii*), (*iii*), (*iv*) is obvious. $\square$

### **Definition 20**

A $$(\mathfrak {X},\mathfrak {F_{p}^*})$$ is called

(i) $\mathfrak {F_{p}^*} T_2$ space a) if for all $x,y \in \mathfrak {X}, x \ne y$ there exists a $PF\mathfrak {F_{p}^*}OS, U=(\mu _{U},\nu _{U}),V=(\mu _{V},\nu _{V}) \in \mathfrak {F_{p}^*}$ such that $\mu _{U}(x)=1,\nu _{U}(x)=0,\mu _{U}(y)=1,\nu _{U}(y)=0$ and $U \cap V = 0_{\mathfrak {X}}.$
(ii) $\mathfrak {F_{p}^*} T_2$ space b) if for all $x,y \in \mathfrak {X}, x \ne y$ there exists a $PF\mathfrak {F_{p}^*}OS, U=(\mu _{U},\nu _{U}),V=(\mu _{V},\nu _{V}) \in \mathfrak {F_{p}^*}$ such that $\mu _{U}(x)=1,\nu _{U}(x)=0,\mu _{U}(y)>0,\nu _{U}(y)=0$ and $U \cap V = (0,\beta )$ where $\beta \in (0,1].$
(iii) $\mathfrak {F_{p}^*} T_2$ space c) if for all $x,y \in \mathfrak {X}, x \ne y$ there exists a $PF\mathfrak {F_{p}^*}OS, U=(\mu _{U},\nu _{U}),V=(\mu _{V},\nu _{V}) \in \mathfrak {F_{p}^*}$ such that $\mu _{U}(x)>0,\nu _{U}(x)=0,\mu _{U}(y)=1,\nu _{U}(y)=0$ and $U \cap V = (0,\beta )$ where $\beta \in (0,1].$
(iv) $\mathfrak {F_{p}^*} T_2$ space d) if for all $x,y \in \mathfrak {X}, x \ne y$ there exists a $PF\mathfrak {F_{p}^*}OS, U=(\mu _{U},\nu _{U}),V=(\mu _{V},\nu _{V}) \in \tau _{{p}cel}$ such that $\mu _{U}(x)>0,\nu _{U}(x)=0,\mu _{U}(y)>0,\nu _{U}(y)=0$ and $U \cap V = (0,\beta )$ where $\beta \in (0,1].$

**Fig. 4:**
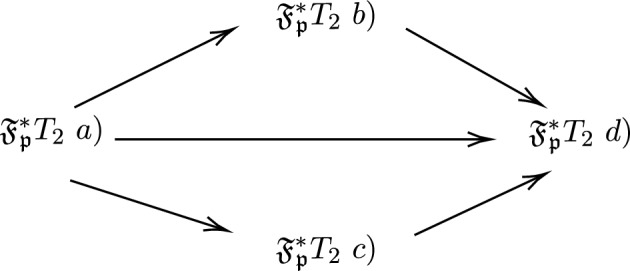
This diagram depicts the implication of $\mathfrak {F_{p}^*} T_2$ space.

### **Proposition 12**

*Let* $(\mathfrak {X},\mathfrak {F_{p}^*})$ *be a* $PF\mathfrak {F_{p}^*}SS$. *Then the following implications hold and it is given in Fig.* 4

### *Proof*

To prove $\mathfrak {F_{p}^*} T_{2}\ a) \Rightarrow \mathfrak {F_{p}^*} T_{2}\ b)$. Let $(\mathfrak {X},\mathfrak {F_{p}^*})$ be a $\mathfrak {F_{p}^*} T_{2} \ a)$ by definition of $PF_{cel} T_{2} \ a)$ for all $x,y \in \mathfrak {X} x \ne y$ there exists $U=(\mu _{U},\nu _{U}),V=(\mu _{V},\nu _{V}) \in \mathfrak {F_{p}^*}$ such that $\mu _{U}(x)=1, \nu _{U}(x)=0, \mu _{U}(y)=1, \mu _{U}(y)=0$ and $U\cap V = 0_{\mathfrak {X}}$ implies $\mu _{U}(x)=1, \nu _{U}(x)=0, \mu _{U}(y)>0, \mu _{U}(y)=0$ and $U\cap V = (0,\beta )$ where $\beta \in (0,1]$ which is $PF_{cel} T_{2}\ b)$. Hence $\mathfrak {F_{p}^*} T_{2}\ a) \Rightarrow \mathfrak {F_{p}^*}T_{2}\ b)$. Similarly, $\mathfrak {F_{p}^*} T_{2}\ a) \Rightarrow \mathfrak {F_{p}^*} T_{2}\ c)$

$$\mathfrak {F_{p}^*} T_{2}\ a) \Rightarrow \mathfrak {F_{p}^*} T_{2}\ d)$$,

$$\mathfrak {F_{p}^*}T_{2}\ b) \Rightarrow \mathfrak {F_{p}^*} T_{2}\ c)$$,

$$\mathfrak {F_{p}^*} T_{2}\ c) \Rightarrow \mathfrak {F_{p}^*} T_{2}\ d)$$. $$\square$$

## Pythagorean fuzzy $\mathfrak {F_{p}^*}$ fraction dense space (PF$\mathfrak {F_{p}^*}$FDS)

In this section we define Pythagorean fuzzy $\mathfrak {F_{p}^*}$ fraction dense space using the Pythagorean fuzzy $\mathfrak {F_{p}^*}$ structure space.

### **Definition 21**

A PF$\mathfrak {F_{p}^*}$SS ($\mathfrak {X}, \mathfrak {F_{p}^*}$) is called Pythagorean fuzzy $\mathfrak {F_{p}^*}$ fraction dense space (PF$\mathfrak {F_{p}^*}$FDS) if for each $PF\mathfrak {F_{p}}^*OS$ *M* in $(\mathfrak {X}, \mathfrak {F_{p}^*})$, $cl_{\mathfrak {F_{p}^*}}(M)= cl_{\mathfrak {F_{p}^*}}(F)$ where *F* is a $PF\mathcal {G^*}CS$ in $( \mathfrak {X}, \mathfrak {F_{p}^*} )$.

### *Example 6*

Consider the frame $\mathfrak {F}=\{\mathfrak {X}, \emptyset , \{b,c\},\{c,a\},\{a\}\}$. The PFFs $\mathcal {P},\mathcal {Q},\mathcal {R}$ are defined as $\mathcal {P}=\{(\mu _{\mathcal {P}}(x),\nu _{\mathcal {P}}(x))|x \in \mathfrak {F}\}$, $\mathcal {Q}=\{(\mu _{\mathcal {Q}}(x),\nu _{\mathcal {Q}}(x))|x \in \mathfrak {F}\}$, $\mathcal {R}=\{(\mu _{\mathcal {R}}(x),\nu _{\mathcal {R}}(x))$

$$|x \in \mathfrak {F}\}$$ where,$$F_{\mathcal {P}}(\mathfrak {X})=\mu _{\mathcal {P}}(\emptyset )=1_\mathfrak {X}, \mu _{P}(\{b,c\})=0.2, \mu _{P}(\{c,a\})=0.4, \mu _{P}(\{c\})=0.5$$

$$\nu _{\mathcal {P}}(\mathfrak {X})=\nu _{\mathcal {P}}(\emptyset )=0_\mathfrak {X}, \nu _{P}(\{b,c\})=0.7, \nu _{P}(\{c,a\})=0.7, \nu _{P}(\{c\})=0.3$$

$$F_{\mathcal {Q}}(\mathfrak {X})=\mu _{\mathcal {Q}}(\emptyset )=1_\mathfrak {X}, \mu _{Q}(\{b,c\})=0.2, \mu _{Q}(\{c,a\})=0.5, \mu _{Q}(\{c\})=0.3$$

$$\nu _{\mathcal {Q}}(\mathfrak {X})=\nu _{\mathcal {Q}}(\emptyset )=0_\mathfrak {X}, \nu _{Q}(\{b,c\})=0.7, \nu _{Q}(\{c,a\})=0.5, \nu _{Q}(\{c\})=0.6$$

$$F_{\mathcal {R}}(\mathfrak {X})=\mu _{\mathcal {R}}(\emptyset )=1_\mathfrak {X}, \mu _{R}(\{b,c\})=0.6, \mu _{R}(\{c,a\})=0.4, \mu _{R}(\{c\})=0.3$$

$$\nu _{\mathcal {R}}(\mathfrak {X})=\nu _{\mathcal {R}}(\emptyset )=0_{\mathfrak {X}}, \nu _{R}(\{b,c\})=0.3, \nu _{R}(\{c,a\})=0.5, \nu _{R}(\{c\})=0.3$$.

Therefore the collection of PFFs $\mathfrak {F_{p}^*} =\{0_{\mathfrak {X}},1_{\mathfrak {X}},\mathcal {P},\mathcal {Q},\mathcal {R}\}$. Then the structure $(\mathfrak {X}, \mathfrak {F_{p}^*})$ is a PF$\mathfrak {F_{p}^*}$SS. Let $\mathcal {S},\mathcal {T}$ are the PF$\mathcal {G^*}$CS in $(\mathfrak {X}, \mathfrak {F_{p}^*})$ which is defined as,

$$\mu _{S}(\{b,c\})=0.3, \mu _{S}(\{c,a\})=0.5, \mu _{S}(\{c\})=0.3$$

$$\nu _{S}(\{b,c\})=0.6, \nu _{S}(\{c,a\})=0.5, \nu _{S}(\{c\})=0.4$$

$$\mu _{T}(\{b,c\})=0.3, \mu _{T}(\{c,a\})=0.3, \mu _{T}(\{c\})=0.3$$

$$\nu _{T}(\{b,c\})=0.6, \nu _{T}(\{c,a\})=0.5, \nu _{T}(\{c\})=0.$$

Then the $cl_{\mathfrak {F_{p}^*}}(PF\mathcal {G^*}CS)$ = $cl_{\mathfrak {F_{p}^*}}(PF\mathfrak {F_{p}^*}OS)$ is PF$\mathfrak {F_{p}^*}$S. Therefore, PF$\mathfrak {F_{p}^*}$SS $(\mathfrak {X}, \mathfrak {F_{p}^*})$ is called PF$\mathfrak {F_{p}^*}$FDS.

### **Proposition 13**

*A PF*$\mathfrak {F_{p}^*}$*SS* $(\mathfrak {X}, \mathfrak {F_{p}^*})$ *is a PF*$\mathfrak {F_{p}^*}$*DS if and only if for each PF*$\mathfrak {F_{p}^*}$*RCS* *F* *in* $(\mathfrak {X}, \mathfrak {F_{p}^*})$, $F= cl_{\mathfrak {F_{p}^*}}(N)$ *where* *N is a PF*$\mathcal {G}^*$*CS in* $(\mathfrak {X}, \mathfrak {F_{p}^*})$.

### *Proof*

Let *F* be a PF$\mathfrak {F_{p}^*}$RCS in $(\mathfrak {X}, \mathfrak {F_{p}^*})$. Then $cl_{\mathfrak {F_{p}^*}}(int_{\mathfrak {F_{p}^*}}(F))=F$ in $( \mathfrak {X}, \mathfrak {F_{p}^*} )$. Let $E=int_{{\mathfrak {F}_{p}^*}}(F)$. Then *K* is PF$\mathfrak {F_{p}^*}$OS in $( \mathfrak {X}, \mathfrak {F_{p}^*} )$. Since $( \mathfrak {X}, \mathfrak {F_{p}^*} )$ is a PF$\mathfrak {F_{p}^*}$FDS, $cl_{\mathfrak {F_{p}^*}}(K)=cl_{\mathfrak {F_{p}^*}}(N)$ where *N* is a PF$\mathcal {G^*}$CS in $(\mathfrak {X}, \mathfrak {F_{p}^*})$. Thus $F=cl_{\mathfrak {F_{p}^*}}(int_{\mathfrak {F_{p}^*}}(F))=cl_{\mathfrak {F_{p}^*}}(K)=cl_{\mathfrak {F_{p}^*}}(N)$ and $F=cl_{\mathfrak {F_{p}^*}}(K)$ in $(\mathfrak {X}, \mathfrak {F_{p}^*})$. Conversely, let *T* be a PF$\mathfrak {F_{p}^*}$OS in $(\mathfrak {X}, \mathfrak {F_{p}^*})$. Then $cl_{\mathfrak {F_{p}^*}}(T)$ is a PF $\mathfrak {F_{p}^*}$ RCS in $(\mathfrak {X}, \mathfrak {F_{p}^*})$. By Proposition 4.10 $cl_{\mathfrak {F_{p}^*}}(T)=cl_{\mathfrak {F_{p}^*}}(N)$ where *K* is a PF$\mathcal {G^*}$CS in $(\mathfrak {X}, \mathfrak {F_{p}^*})$ and then $(\mathfrak {X}, \mathfrak {F_{p}^*})$ is PF$\mathfrak {F_{p}^*}$FDS. $\square$

### **Proposition 14**

*If *$(\mathfrak {X}, \mathfrak {F_{p}^*})$ *is a PF*$\mathfrak {F_{p}^*}$*FDS and* *L* *is a PF*$\mathfrak {F_{p}^*}$*ROS in* $(\mathfrak {X}, \mathfrak {F_{p}^*})$ *then* $L=int_{\mathfrak {F_{p}^*}}(R)$ *where* *R* *is a PF*$\mathcal {G^*}OS$ *in* $(\mathfrak {X}, \mathfrak {F_{p}^*})$.

### *Proof*

Let *L* is a PF$\mathfrak {F_{p}^*}$ROS in $(\mathfrak {X}, \mathfrak {F_{p}^*})$. $L^c$ is a PF$\mathfrak {F_{p}^*}$RCS in $(\mathfrak {X}, \mathfrak {F_{p}^*})$. Since $(\mathfrak {X}, \mathfrak {F_{p}^*})$ is a PF$\mathfrak {F_{p}^*}$FDS by Proposition 6.3 $L^c= cl_{\mathfrak {F_{p}^*}}(K)$ where *K* is a PF$\mathcal {G^*}$CS in $(\mathfrak {X}, \mathfrak {F_{p}^*})$. Then $L=[cl_{\mathfrak {F_{p}^*}}(K)]^c=int_{\mathfrak {F_{p}^*}}(K^c)$ by Proposition 4.11. Let $R=K^c$ where *K* is a PF$\mathcal {G^*}$OS in $(\mathfrak {X}, \mathfrak {F_{p}^*})$. Hence $L=int_{\mathfrak {F_{p}^*}}(R)$ where *R* is a PF$\mathcal {G^*}$OS in $(\mathfrak {X}, \mathfrak {F_{p}^*})$. $\square$

### **Proposition 15**

*If* *K is a PF*$\mathfrak {F_{p}^*}$*OS in PF*$\mathfrak {F_{p}^*}$*FDS* $(\mathfrak {X}, \mathfrak {F_{p}^*})$, *then there exists a PF*$\mathcal {G^*}$*CS* *G in* $(\mathfrak {X}, \mathfrak {F_{p}^*})$ *such that* $K \subseteq cl_{\mathfrak {F_{p}^*}}(K)$.

### *Proof*

Let *K* be a PF$\mathfrak {F_{p}^*}$OS in $(\mathfrak {X}, \mathfrak {F_{p}^*})$. Since $(\mathfrak {X}, \mathfrak {F_{p}^*})$ is a PF$\mathfrak {F_{p}^*}$FDS. $cl_{\mathfrak {F_{p}^*}}(K)=cl_{\mathfrak {F_{p}^*}}(S)$ where *S* is a PF$\mathfrak {F_{p}^*}$CS in $(\mathfrak {X}, \mathfrak {F_{p}^*})$. $G \subseteq cl_{\mathfrak {F_{p}^*}}(K)$ implies $G \subseteq cl_{\mathfrak {F_{p}^*}}(S)$. $\square$

### **Proposition 16**

*If* *K is a PF*$\mathfrak {F_{p}^*}$*OS in a PF*$\mathfrak {F_{p}^*}$*DS in* $(\mathfrak {X}, \mathfrak {F_{p}^*})$,* then there exists a PF*$\mathcal {G^*}$*CS S in *$(\mathfrak {X}, \mathfrak {F_{p}^*})$ *such that *$K \subseteq cl_{\mathfrak {F_{p}^*}}(N)$.

### *Proof*

Let *K* be a PF$\mathfrak {F_{p}^*}$OS in $(\mathfrak {X}, \mathfrak {F_{p}^*})$. Now $K \subseteq cl_{\mathfrak {F_{p}^*}}(K)$ in $(\mathfrak {X}, \mathfrak {F_{p}^*})$. Since $(\mathfrak {X}, \mathfrak {F_{p}^*})$ is a PF$\mathfrak {F_{p}^*}$FDS, $cl_{\mathfrak {F_{p}^*}}(K)=cl_{\mathfrak {F_{p}^*}}(S)$. Then *K* is PF$\mathcal {G^*}$CS in $(\mathfrak {X}, \mathfrak {F_{p}^*})$. Thus there exists a PF$\mathcal {G^*}$CS *K* in $(\mathfrak {X}, \mathfrak {F_{p}^*})$ such that $K \subseteq cl_{\mathfrak {F_{p}^*}}(N)$. $\square$

### **Proposition 17**

*If* *K is a PF*$\mathfrak {F_{p}^*}$*OS in PF*$\mathfrak {F_{p}^*}$*FDS in *$(\mathfrak {X}, \mathfrak {F_{p}^*})$, *then there exists PF*$\mathcal {G^*}$*CS* *G* *and* *S in* $(\mathfrak {X}, \mathfrak {F_{p}^*})$ *such that* $G \subseteq cl_{\mathfrak {F_{p}^*}}(K) \subseteq cl_{\mathfrak {F_{p}^*}}(S)$.

### *Proof*

Let *K* be a PF$\mathfrak {F_{p}^*}$OS in $(\mathfrak {X}, \mathfrak {F_{p}^*})$. Since $(\mathfrak {X}, \mathfrak {F_{p}^*})$ is a PF$\mathfrak {F_{p}^*}$FDS, by Proposition 6.5, there exists a PF$\mathfrak {F_{p}^*}$CS *G* in $(\mathfrak {X}, \mathfrak {F_{p}^*})$ such that $G \subseteq cl_{\mathfrak {F_{p}^*}}(K)$. Also by Proposition 6.6, there exists a PF$\mathcal {G^*}$CS *E* in $(\mathfrak {X}, \mathfrak {F_{p}^*})$ such that $K \subseteq cl_{\mathfrak {F_{p}^*}}(S)$. Then $G \subseteq cl_{\mathfrak {F_{p}^*}}(K) \subseteq cl_{\mathfrak {F_{p}^*}}({cl_{\mathfrak {F_{p}^*}}(S)})$. Since $cl_{\mathfrak {F_{p}^*}}({cl_{\mathfrak {F_{p}^*}}(S)})=cl_{\mathfrak {F_{p}^*}}(S)$ in $(\mathfrak {X}, \mathfrak {F_{p}^*})$, for a PF$\mathfrak {F_{p}^*}$OS *K* in $(\mathfrak {X}, \mathfrak {F_{p}^*})$, $G \subseteq cl_{\mathfrak {F_{p}^*}}(K) \subseteq cl_{\mathfrak {F_{p}^*}}(S)$. $\square$

### **Proposition 18**

*If* *Q* *is a PF*$\mathfrak {F_{p}^*}$*CS in PF*$\mathfrak {F_{p}^*}$*FDS* $(\mathfrak {X}, \mathfrak {F_{p}^*})$, *then there exists a PF*$\mathcal {G^*}$*OS R* in $(\mathfrak {X}, \mathfrak {F_{p}^*})$ *such that* $int_{\mathfrak {F_{p}^*}}(Q)\subseteq R$.

### *Proof*

Let *Q* is a PF$\mathfrak {F_{p}^*}$CS. Then $Q^c$ is a PF$\mathfrak {F_{p}^*}$OS in $(\mathfrak {X}, \mathfrak {F_{p}^*})$. Since $(\mathfrak {X}, \mathfrak {F_{p}^*})$ is a PF$\mathfrak {F_{p}^*}$FDS by Proposition 6.5 there exists a PF$\mathcal {G^*}$CS *G* in $(\mathfrak {X}, \mathfrak {F_{p}^*})$, $G \subseteq cl_{\mathfrak {F_{p}^*}}(Q^c)$. Then, $G \subseteq [int_{\mathfrak {F_{p}^*}}(Q)]^c$ by Proposition 4.11. This implies that $int_{\mathfrak {F_{p}^*}}(Q)\subseteq G^c$. Let $R = G^c$. Then $R^c$ is a PF$\mathcal {G^*}$OS and $int_{\mathfrak {F_{p}^*}}(Q)\subseteq R$ in $(\mathfrak {X},\mathfrak {F_{p}^*})$. $\square$

### **Proposition 19**

*If* *Q is a PF*$\mathfrak {F_{p}^*}$*CS in PF*$\mathfrak {F_{p}^*}$*FDS *$(\mathfrak {X},\mathfrak {F_{p}^*})$, *then there exists a PF*$\mathcal {G^*}$*OS M* *in *$(\mathfrak {X},\mathfrak {F_{p}^*})$ *such that *$int_{\mathfrak {F_{p}^*}}(M)\subseteq Q$.

### *Proof*

Let *Q* is a PF$\mathfrak {F_{p}^*}$CS in $(\mathfrak {X},\mathfrak {F_{p}^*})$. Then $Q^c$ is PF$\mathfrak {F_{p}^*}$OS in $(\mathfrak {X},\mathfrak {F_{p}^*})$. Since $(\mathfrak {X},\mathfrak {F_{p}^*})$ is PF$\mathfrak {F_{p}^*}$FDS by Proposition 6.6, there exists a PF$\mathcal {G^*}$CS in $(\mathfrak {X},\mathfrak {F_{p}^*})$ such that $Q^c\subseteq cl_{\mathfrak {F_{p}^*}}(K)$. then, $[cl_{\mathfrak {F_{p}^*}}(K)]^c \subseteq Q$ and by Proposition 4.11 $int_{\mathfrak {F_{p}^*}}(K^c) = [cl_{\mathfrak {F_{p}^*}}(K)]^c \subseteq Q$. Let $M=K^c$ and *M* is a PF$\mathcal {G^*}$OS in $(\mathfrak {X},\mathfrak {F_{p}^*})$ and $int_{\mathfrak {F_{p}^*}}(M)\subseteq Q$ in $(\mathfrak {X},\mathfrak {F_{p}^*})$. $\square$

### **Proposition 20**

*If* *Q* *is a PF*${\mathfrak {F_{p}^*}}$*CS in PF*$\mathfrak {F_{p}^*}$*FDS in *$(\mathfrak {X},\mathfrak {F_{p}^*})$, *then there exists PF*$\mathcal {G^*}$*OS* *M* *and* *R in* $(\mathfrak {X},\mathfrak {F_{p}^*})$ *such that* $int_{\mathfrak {F_{p}^*}}(M)\subseteq int_{\mathfrak {F_{p}^*}}(Q)\subseteq R$ *in* $(\mathfrak {X},\mathfrak {F_{p}^*})$.

### *Proof*

Let *Q* be a PF$\mathfrak {F_{p}^*}$CS in $(\mathfrak {X},\mathfrak {F_{p}^*})$. Since $(\mathfrak {X},\mathfrak {F_{p}^*})$ is PF$\mathfrak {F_{p}^*}$FDS, by Proposition 6.8 there exists a PF$\mathcal {G^*}$OS *R* in $(\mathfrak {X},\mathfrak {F_{p}^*})$ such that $int_{\mathfrak {F_{p}^*}}(Q)\subseteq R$. Also by Proposition 6.9, there exists a PF$\mathcal {G^*}$OS *M* in $(\mathfrak {X},\mathfrak {F_{p}^*})$ such that $int_{\mathfrak {F_{p}^*}}(M)\subseteq Q$, then $int_{\mathfrak {F_{p}^*}}(int_{\mathfrak {F_{p}^*}}(M))\subset int(Q) \subseteq R$ in $(\mathfrak {X},\mathfrak {F_{p}^*})$. This implies that $int_{\mathfrak {F_{p}^*}}(M) \subseteq int_{\mathfrak {F_{p}^*}}(Q) \subseteq R$ in $(\mathfrak {X},\mathfrak {F_{p}^*})$. $\square$

### **Proposition 21**

*If* *L is a PF*$\mathfrak {F_{p}^*}$*ROS in a PF*$\mathfrak {F_{p}^*}$*FDS* $(\mathfrak {X},\mathfrak {F_{p}^*})$ *then there exists a PF*$\mathcal {G^*}$*OS* *R with *$int_{\mathfrak {F_{p}^*}}cl_{\mathfrak {F_{p}^*}}(R)\ne 0_{\mathfrak {X}}$ *in* $(\mathfrak {X},\mathfrak {F_{p}^*})$ *such that* $L \subseteq R$.

### *Proof*

Let *L* be a PF$\mathfrak {F_{p}^*}$ROS in $(\mathfrak {X},\mathfrak {F_{p}^*})$. Since $(\mathfrak {X},\mathfrak {F_{p}^*})$ is a PF$\mathfrak {F_{p}^*}$FDS, by Proposition 6.4, there exists a PF$\mathcal {G^*}$OS *R* in $(\mathfrak {X},\mathfrak {F_{p}^*})$ such that $L=int_{\mathfrak {F_{p}^*}}(R)$. Now $int_{\mathfrak {F_{p}^*}}(R)\subseteq int_{\mathfrak {F_{p}^*}}(cl_{\mathfrak {F_{p}^*}}(R))$. This implies $L= int_{\mathfrak {F_{p}^*}}(cl_{\mathfrak {F_{p}^*}}(R))$ and thus $int_{\mathfrak {F_{p}^*}}(cl_{\mathfrak {F_{p}^*}}(R)) \ne 0_{\mathfrak {X}}$. Thus there is a PF$\mathcal {G^*}$OS *R* with $int_{\mathfrak {F_{p}^*}}(cl_{\mathfrak {F_{p}^*}}(R)) \ne 0_{\mathfrak {X}}$ in $(\mathfrak {X},\mathfrak {F_{p}^*})$ such that $L \subseteq R$. $\square$

### **Corollary 1**

*If* *L is a PF*$\mathfrak {F_{p}^*}$*ROS in a PF*$\mathfrak {F_{p}^*}$*FDS* $(\mathfrak {X},\mathfrak {F_{p}^*})$, *then there exists a PFsWDS* *R* in $(\mathfrak {X},\mathfrak {F_{p}^*})$ *such that *$L\subseteq R$.

### *Proof*

Let *L* be a PF$\mathfrak {F_{p}^*}$ ROS in $(\mathfrak {X},\mathfrak {F_{p}^*})$.Since $(\mathfrak {X},\mathfrak {F_{p}^*})$ is a PF$\mathfrak {F_{p}^*}$FDS,by Proposition 6.11 there exists a PF$\mathcal {G^*}$OS *R* with $int_{ \mathfrak {F_{p}^*}}(cl_{ \mathfrak {F_{p}^*}}(R)) \ne 0_{\mathfrak {X}}$ in $(\mathfrak {X},\mathfrak {F_{p}^*})$ such that $L \subseteq R$. Now $int_{ \mathfrak {F_{p}^*}}cl_{ \mathfrak {F_{p}^*}}(R)$ $\ne 0_{\mathfrak {X}}$ implies that *R* is a PFsWDS in $(\mathfrak {X},\mathfrak {F_{p}^*})$. $\square$

### **Proposition 22**

*If* *M* *is a PF*$\mathfrak {F_{p}^*}$*RCS in PF*$\mathfrak {F_{p}^*}$*FDS* $(\mathfrak {X},\mathfrak {F_{p}^*})$, *then there exists a PF*$\mathcal {G^*}$*CS* *Q* *in* $(\mathfrak {X},\mathfrak {F_{p}^*})$ *such that* $Q\subseteq M$.

### *Proof*

Let *M* be a PF$\mathfrak {F_{p}^*}$RCS in $(\mathfrak {X},\mathfrak {F_{p}^*})$. Then $M^c$ is a PF$\mathfrak {F_{p}^*}$ROS in $(\mathfrak {X},\mathfrak {F_{p}^*})$. Since $(\mathfrak {X},\mathfrak {F_{p}^*})$ is a PF$\mathfrak {F_{p}^*}$FDS by Proposition 6.11 there exists a PF$\mathcal {G^*}$OS *R* in $( \mathfrak {X}, \mathfrak {F_{p}^*})$ such that $M^c\subseteq R$ then $M \subseteq Q^c$. Let $Q= R^c$ Let *Q* is a PF$\mathcal {G^*}$CS in $( \mathfrak {X}, \mathfrak {F_{p}^*})$. Hence there exists a PF$\mathcal {G^*}$CS *Q* in $(\mathfrak {X}, \mathfrak {F_{p}^*})$ such that $Q \subseteq M$. $\square$

### **Corollary 2**

*If* *M is a PF*$\mathfrak {F_{p}^*}$*RCS in PF*$\mathfrak {F_{p}^*}$*FDS* $(\mathfrak {X},\mathfrak {F_{p}^*})$, *then there exists a PFcs-DS* *Q in* $(\mathfrak {X}, \mathfrak {F_{p}^*})$ *such that* $Q \subseteq M$.

### *Proof*

Let *M* be a PF$\mathfrak {F_{p}^*}$RCS in $(\mathfrak {X}, \mathfrak {F_{p}^*})$. Then $M^c$ is a PF$\mathfrak {F_{p}^*}$ROS in $(\mathfrak {X}, \mathfrak {F_{p}^*})$. Since $(\mathfrak {X}, \mathfrak {F_{p}^*})$ is a PF$\mathfrak {F_{p}^*}$FDS by Corollary 6.12, there exists a PFsWDS *R* in $(\mathfrak {X}, \mathfrak {F_{p}^*})$ such that $M^c\subseteq R$ in $(\mathfrak {X}, \mathfrak {F_{p}^*})$. Then $R^c \subseteq M$. Let $Q \subseteq R^c$. Then *Q* is a PFcs-DS in $(\mathfrak {X}, \mathfrak {F_{p}^*})$ and $Q \subseteq M$. $\square$

### **Proposition 23**

*If* $(\mathfrak {X}, \mathfrak {F_{p}^*})$ *is a PF*$\mathfrak {F_{p}^*}$*FDS, then there exists a PF*$\mathcal {G^*}$*CS* *K* *and PF*$\mathcal {G^*}$*OS* *R* *in* $(\mathfrak {X}, \mathfrak {F_{p}^*})$ *such that *$K \subseteq cl_{\mathfrak {F_{p}^*}}(R)$.

### *Proof*

Let *L* be a PF$\mathfrak {F_{p}^*}$ROS in $(\mathfrak {X}, \mathfrak {F_{p}^*})$. Since $(\mathfrak {X}, \mathfrak {F_{p}^*})$ is PF$\mathfrak {F_{p}^*}$FDS by Proposition 6.11 there exists a PF$\mathcal {G^*}$OS *R* in $(\mathfrak {X}, \mathfrak {F_{p}^*})$ such that $L\subseteq R$ and then $cl_{\mathfrak {F_{p}^*}}(L)\subseteq cl_{\mathfrak {F_{p}^*}}(R)$ in $(\mathfrak {X}, \mathfrak {F_{p}^*})$. Since a PF$\mathfrak {F_{p}^*}$ROS is a PF$\mathfrak {F_{p}^*}$OS in $(\mathfrak {X}, \mathfrak {F_{p}^*})$, by Proposition 6.5. there exists a PF$\mathcal {G^*}$CS *K* in $(\mathfrak {X}, \mathfrak {F_{p}^*})$ such that $K \subseteq cl_{\mathfrak {F_{p}^*}}(L)$. Then $K \subseteq cl_{\mathfrak {F_{p}^*}}(L)\subseteq cl_{\mathfrak {F_{p}^*}}(R)$ and thus $K \subseteq cl_{\mathfrak {F_{p}^*}}(R)$ in $(\mathfrak {X}, \mathfrak {F_{p}^*})$. $\square$

The following Propositions from Proposition 6.16 to Proposition 6.23 shows that PF$\mathfrak {F_{p}^*}$CS are not PFnWDS and the PF$\mathfrak {F_{p}^*}$OS are not PFDS in PF$\mathfrak {F_{p}^*}$FDS.

### **Proposition 24**

*If* *Q is a PF*$\mathfrak {F_{p}^*}$*CS in PF*$\mathfrak {F_{p}^*}$*FDS* $(\mathfrak {X}, \mathfrak {F_{p}^*})$, *then Q* *is not a PFnWDS in* $(\mathfrak {X}, \mathfrak {F_{p}^*})$.

### *Proof*

Let *Q* be a PF$\mathfrak {F_{p}^*}$CS in $(\mathfrak {X}, \mathfrak {F_{p}^*})$. Since $(\mathfrak {X}, \mathfrak {F_{p}^*})$ is a PF$\mathfrak {F_{p}^*}$FDS,by Proposition 6.9 there exists a PF$\mathfrak {F_{p}^*}$OS *M* in $(\mathfrak {X}, \mathfrak {F_{p}^*})$ such that $int_{\mathfrak {F_{p}^*}}(M)\subseteq Q$. Then $int_{\mathfrak {F_{p}^*}}(Q) \ne 0_{\mathfrak {X}}$ and $int_{\mathfrak {F_{p}^*}}(Q)\subseteq int_{\mathfrak {F_{p}^*}}(cl_{\mathfrak {F_{p}^*}}(Q))$ implies $int_{\mathfrak {F_{p}^*}}(cl_{\mathfrak {F_{p}^*}}(Q)) \ne 0_{\mathfrak {X}}$ in $(\mathfrak {X}, \mathfrak {F_{p}^*})$. Hence *Q* is not a PFnWDS in $(\mathfrak {X}, \mathfrak {F_{p}^*})$. $\square$

### **Proposition 25**

*If* *K* *is a PF*$\mathfrak {F_{p}^*}$*OS in PF*$\mathfrak {F_{p}^*}$*FDS *$(\mathfrak {X}, \mathfrak {F_{p}^*})$ *then* *K* *is not a PFDS in *$(\mathfrak {X}, \mathfrak {F_{p}^*})$.

### *Proof*

Let *K* is a PF$\mathfrak {F_{p}^*}$OS in $(\mathfrak {X}, \mathfrak {F_{p}^*})$. Suppose that $cl_{\mathfrak {F_{p}^*}}(K)=1_{\mathfrak {X}}$ in $(\mathfrak {X}, \mathfrak {F_{p}^*})$. Then $int_{\mathfrak {F_{p}^*}}(cl_{\mathfrak {F_{p}^*}}(K^c))= [cl_{\mathfrak {F_{p}^*}}(int_{\mathfrak {F_{p}^*}}(K))]^c=[cl_{\mathfrak {F_{p}^*}}(K)]^c=0_{\mathfrak {X}}$. This implies that the PF$\mathfrak {F_{p}^*}$CS $K^c$ is a PFnWDS in the PF$\mathfrak {F_{p}^*}$FDS $(\mathfrak {X}, \mathfrak {F_{p}^*})$, a contradiction by Proposition 6.16. Hence *K* is a not a PFDS in $(\mathfrak {X}, \mathfrak {F_{p}^*})$. $\square$

### **Proposition 26**

*If* *Q* *is a PF*$\mathfrak {F_{p}^*}$*CS in PF*$\mathfrak {F_{p}^*}$*FDS* $(\mathfrak {X}, \mathfrak {F_{p}^*})$ *there exists a PF*$\mathfrak {F_{p}^*}$*RCS K* *in* $(\mathfrak {X}, \mathfrak {F_{p}^*})$ *such that *$K \subseteq Q$.

### *Proof*

Let *Q* is a PF$\mathfrak {F_{p}^*}$CS in $(\mathfrak {X}, \mathfrak {F_{p}^*})$, then $cl_{\mathfrak {F_{p}^*}}(Q)=Q$. Since $(\mathfrak {X}, \mathfrak {F_{p}^*})$ is a PF$\mathfrak {F_{p}^*}$FDS, Proposition 6.16, *Q* is a not a PFnWDS in $(\mathfrak {X}, \mathfrak {F_{p}^*})$ and then $int_{\mathfrak {F_{p}^*}}cl_{\mathfrak {F_{p}^*}}(Q) \ne 0_{\mathfrak {X}}$ in $(\mathfrak {X}, \mathfrak {F_{p}^*})$. Now $int_{\mathfrak {F_{p}^*}}(Q)=int_{\mathfrak {F_{p}^*}}(cl_{\mathfrak {F_{p}^*}}(Q))) \ne 0_{\mathfrak {X}}$ and then there exists a PF$\mathfrak {F_{p}^*}$OS *K* in $(\mathfrak {X}, \mathfrak {F_{p}^*})$ such that $K \subseteq Q$. Then $cl_{\mathfrak {F_{p}^*}}(K)\subseteq cl_{\mathfrak {F_{p}^*}}(Q)= Q$ and $cl_{\mathfrak {F_{p}^*}}(K)$ is a PF$\mathfrak {F_{p}^*}$RCS in $(\mathfrak {X}, \mathfrak {F_{p}^*})$ by Proposition 4.10. Let $K=cl_{\mathfrak {F_{p}^*}}(K)$. Hence there exists a PF$\mathfrak {F_{p}^*}$RCS *K* in $(\mathfrak {X}, \mathfrak {F_{p}^*})$ such that $K \subseteq Q$. $\square$

### **Proposition 27**

*If* *K* *is a PF*$\mathfrak {F_{p}^*}$*OS in a PF*$\mathfrak {F_{p}^*}$*FDS* $(\mathfrak {X}, \mathfrak {F_{p}^*})$ *then there exists a PF*$\mathfrak {F_{p}^*}$*ROS* *L in* $(\mathfrak {X}, \mathfrak {F_{p}^*})$ *such that* $K\subseteq L$.

### *Proof*

Let *K* is a PF$\mathfrak {F_{p}^*}$OS in $(\mathfrak {X}, \mathfrak {F_{p}^*})$. Then $K^c$ is a PF$\mathfrak {F_{p}^*}$CS in $(\mathfrak {X}, \mathfrak {F_{p}^*})$. Since $(\mathfrak {X}, \mathfrak {F_{p}^*})$ is a PF$\mathfrak {F_{p}^*}$FDS by Proposition 6.18 there exists a PF$\mathfrak {F_{p}^*}$RCS *K* in $(\mathfrak {X}, \mathfrak {F_{p}^*})$ such that $K\subseteq K^c$. Then $K^c = L$. Let $L = K^c$ and *L* is a PF$\mathfrak {F_{p}^*}$ROS in $(\mathfrak {X}, \mathfrak {F_{p}^*})$ and $K \subseteq L$. $\square$

### **Proposition 28**

*If K* *is a PFnWDS in PF*$\mathfrak {F_{p}^*}$*FDS* $(\mathfrak {X}, \mathfrak {F_{p}^*})$, *then there exists a PFcs-DS Q* *in* $(\mathfrak {X}, \mathfrak {F_{p}^*})$ *such that *$Q \subseteq cl_{\mathfrak {F_{p}^*}}(K)$.

### *Proof*

Let *K* be a PFnWDS in $(\mathfrak {X}, \mathfrak {F_{p}^*})$ and then $cl_{\mathfrak {F_{p}^*}}(K)$ is a PF$\mathfrak {F_{p}^*}$CS in $(\mathfrak {X}, \mathfrak {F_{p}^*})$. Since $(\mathfrak {X}, \mathfrak {F_{p}^*})$ is a PF$\mathfrak {F_{p}^*}$FDS, by Proposition 6.18 there exists a PF$\mathfrak {F_{p}^*}$RCS *N* in $(\mathfrak {X}, \mathfrak {F_{p}^*})$ such that $N \subseteq cl_{\mathfrak {F_{p}^*}}(K)$. By Corollary 6.14 there exists a PFcs-DS *Q* in $(\mathfrak {X}, \mathfrak {F_{p}^*})$ such that $Q \subseteq N$ and then $Q \subseteq cl_{\mathfrak {F_{p}^*}}(K)$. $\square$

### **Proposition 29**

*If* *K is a PFnWDS in a PF*$\mathfrak {F_{p}^*}$*FDS* $(\mathfrak {X}, \mathfrak {F_{p}^*})$, *then there exists no non-zero PF*$\mathfrak {F_{p}^*}$*RCS* *N in* $(\mathfrak {X}, \mathfrak {F_{p}^*})$ *such that *$K \subseteq cl_{\mathfrak {F_{p}^*}}(K)$.

### *Proof*

Let *K* is a PFnWDS in $(\mathfrak {X}, \mathfrak {F_{p}^*})$ and $int_{\mathfrak {F_{p}^*}}(cl_{\mathfrak {F_{p}^*}}(K))=0_{\mathfrak {X}}$. Since $(\mathfrak {X}, \mathfrak {F_{p}^*})$ is a PF$\mathfrak {F_{p}^*}$FDS by Proposition 6.18. there exists a PF$\mathfrak {F_{p}^*}$RCS *N* in $(\mathfrak {X}, \mathfrak {F_{p}^*})$ such that $N \subseteq cl_{\mathfrak {F_{p}^*}}(K)$, then $int_{\mathfrak {F_{p}^*}}(N) \subseteq int_{\mathfrak {F_{p}^*}}( cl_{\mathfrak {F_{p}^*}}(K))=0_{\mathfrak {X}}$ and $int_{\mathfrak {F_{p}^*}}(N)=0_{\mathfrak {X}}$. This implies that $cl_{\mathfrak {F_{p}^*}}(int_{\mathfrak {F_{p}^*}}(N))=cl_{\mathfrak {F_{p}^*}}(0_{\mathfrak {X}})=0_{\mathfrak {X}}$ and $N=0_{\mathfrak {X}}$. Thus, there exists no non-zero PF$\mathfrak {F_{p}^*}$RCS *K* in $(\mathfrak {X}, \mathfrak {F_{p}^*})$ such that $N \subseteq cl_{\mathfrak {F_{p}^*}}(K)$. $\square$

### **Proposition 30**

*Let* *K* *is a PF*$\mathfrak {F_{p}^*}$*FDS* $(\mathfrak {X}, \mathfrak {F_{p}^*})$, *then there exists a PF*$\mathcal {G^*}$*OS* *R in* $(\mathfrak {X}, \mathfrak {F_{p}^*})$ *such that* $int_{\mathfrak {F_{p}^*}}(R)\subseteq K$.

### *Proof*

Let *K* be a PF$\mathfrak {F_{p}^*}$ROS in $(\mathfrak {X}, \mathfrak {F_{p}^*})$. Then $int_{\mathfrak {F_{p}^*}}(cl_{\mathfrak {F_{p}^*}}(K))=K$ in $(\mathfrak {X}, \mathfrak {F_{p}^*})$. Now $cl_{\mathfrak {F_{p}^*}}(K)$ is a PF$\mathfrak {F_{p}^*}$CS in $(\mathfrak {X}, \mathfrak {F_{p}^*})$. Since $(\mathfrak {X}, \mathfrak {F_{p}^*})$ is a PF$\mathfrak {F_{p}^*}$FDS, by Proposition 6.9, there exists a PF$\mathcal {G^*}$OS *R* in $(\mathfrak {X}, \mathfrak {F_{p}^*})$ such that $int_{ \mathfrak {F_{p}^*}}(R)\subseteq cl_{\mathfrak {F_{p}^*}}(K)$. Then $int_{\mathfrak {F_{p}^*}}(int_{\mathfrak {F_{p}^*}}(R))\subseteq int_{\mathfrak {F_{p}^*}}(cl_{\mathfrak {F_{p}^*}}(K))$ and $int_{\mathfrak {F_{p}^*}}(R)\subseteq K$ in $(\mathfrak {X}, \mathfrak {F_{p}^*})$. $\square$

### **Proposition 31**

*If* *K is a PF*$\mathfrak {F_{p}^*}$*ROS in a PF*$\mathfrak {F_{p}^*}$*FDS *$(\mathfrak {X}, \mathfrak {F_{p}^*})$, *then there exists a PF*$\mathcal {G^*}$*OS* *R and* *T in* $(\mathfrak {X}, \mathfrak {F_{p}^*})$ *such that* $int_{\mathfrak {F_{p}^*}}(T)\subseteq K \subseteq R$.

### *Proof*

Let *K* be a PF$\mathfrak {F_{p}^*}$ROS in $(\mathfrak {X}, \mathfrak {F_{p}^*})$. Since $(\mathfrak {X}, \mathfrak {F_{p}^*})$ is a PF$\mathfrak {F_{p}^*}$FDS, by Proposition 6.11, there exists a PF$\mathcal {G^*}$OS in $(\mathfrak {X}, \mathfrak {F_{p}^*})$ such that $K \subseteq R$. Also by Proposition 6.22, there exists a PF$\mathcal {G^*}$OS *T* in $(\mathfrak {X}, \mathfrak {F_{p}^*})$ such that $int_{\mathfrak {F_{p}^*}}(T)\subseteq K$. Thus $int_{\mathfrak {F_{p}^*}}(T)\subseteq K \subseteq R$ in $(\mathfrak {X}, \mathfrak {F_{p}^*})$. $\square$

## Pythagorean fuzzy $\mathfrak {F_{p}^*}$ fraction dense space and Pythagorean fuzzy $\mathcal {P^*}$ space (PF$\mathcal {P^*}$S)

In this section Pythagorean fuzzy $\mathcal {P^*}$ space is defined and it is proved that PF$\mathfrak {F_{p}^*}$RCS are PF$\mathcal {G^*}$CS, also PF$\mathfrak {F_{p}^*}$ROS are PF$\mathcal {G^*}$OS in PF$\mathfrak {F_{p}^*}$FDS and PF$\mathcal {P^*}$S.

### **Definition 22**

A PF$\mathfrak {F_{p}^*}$FDS $(\mathfrak {X}, \mathfrak {F_{p}^*})$ is called PF$\mathcal {P^*}$S if each PF$\mathcal {G^*}$OS in $(\mathfrak {X}, \mathfrak {F_{p}^*})$ is PF$\mathfrak {F_{p}^*}$OS in $(\mathfrak {X}, \mathfrak {F_{p}^*})$.

### **Proposition 32**

*If* *F is a PF*$\mathfrak {F_{p}^*}$ *RCS in a PF*$\mathfrak {F_{p}^*}$*FDS and PF*$\mathcal {P^*}$*S* $(\mathfrak {X}, \mathfrak {F_{p}^*})$, *then F* *is a PF*$\mathcal {G^*}$*CS in* $(\mathfrak {X}, \mathfrak {F_{p}^*})$.

### *Proof*

Let *F* be a PF$\mathfrak {F_{p}^*}$RCS in $(\mathfrak {X}, \mathfrak {F_{p}^*})$. Since $(\mathfrak {X}, \mathfrak {F_{p}^*})$ is a PF$\mathfrak {F_{p}^*}$FDS, by Proposition 6.3. $F =cl_{\mathfrak {F_{p}^*}}(K)$ where *K* is a PF$\mathcal {G^*}$CS in $(\mathfrak {X}, \mathfrak {F_{p}^*})$. Since $(\mathfrak {X}, \mathfrak {F_{p}^*})$ is a PF$\mathcal {P^*}$S, PF$\mathcal {G^*}$CS *K* is a PF$\mathfrak {F_{p}^*}$CS and then $cl_{\mathfrak {F_{p}^*}}(K)=K$ in $(\mathfrak {X}, \mathfrak {F_{p}^*})$. Hence PF$\mathfrak {F_{p}^*}$RCS *F* is a PF$\mathcal {G^*}$CS in $(\mathfrak {X}, \mathfrak {F_{p}^*})$. $\square$

### **Corollary 3**

*If* *K is PF*$\mathfrak {F_{p}^*}$*ROS in PF*$\mathfrak {F_{p}^*}$*FDS and PF*$\mathcal {P^*}$*S* $(\mathfrak {X}, \mathfrak {F_{p}^*})$, *then* *K* *is a PF*$\mathcal {G^*}$*OS in* $(\mathfrak {X}, \mathfrak {F_{p}^*})$.

### *Proof*

Let *K* be a PF$\mathfrak {F_{p}^*}$ROS in $(\mathfrak {X}, \mathfrak {F_{p}^*})$. Then $K^c$ is a PF$\mathfrak {F_{p}^*}$CS in $(\mathfrak {X}, \mathfrak {F_{p}^*})$. Since $(\mathfrak {X}, \mathfrak {F_{p}^*})$ is a PF$\mathfrak {F_{p}^*}$FDS, by Proposition 7.2. $K^c$ is PF$\mathcal {G^*}$CS in $(\mathfrak {X}, \mathfrak {F_{p}^*})$ and thus *K* is a PF$\mathcal {G^*}$OS in $(\mathfrak {X}, \mathfrak {F_{p}^*})$. $\square$

### **Proposition 33**

*If* *F* *is a PF*$\mathcal {G^*}$*CS in PF*$\mathfrak {F_{p}^*}$*FDS and PF*$\mathcal {P^*}$*S* $(\mathfrak {X}, \mathfrak {F_{p}^*})$, *then* *F is a PFsWDS in* $(\mathfrak {X}, \mathfrak {F_{p}^*})$.

### *Proof*

Let *F* be a PF$\mathcal {G^*}$CS in $(\mathfrak {X}, \mathfrak {F_{p}^*})$. Since $(\mathfrak {X}, \mathfrak {F_{p}^*})$ is a PF$\mathcal {P^*}$S, the PF$\mathcal {G^*}$CS, *F* is a PF$\mathfrak {F_{p}^*}$CS and then by Proposition 6.16 *F* is not a PFnWDS in $(\mathfrak {X}, \mathfrak {F_{p}^*})$. Thus $int_{\mathfrak {F_{p}^*}}(cl_{\mathfrak {F_{p}^*}}(F)) \ne 0_{\mathfrak {X}}$ in $(\mathfrak {X}, \mathfrak {F_{p}^*})$. Hence *F* is a PFsWDS in $(\mathfrak {X}, \mathfrak {F_{p}^*})$. $\square$

### **Corollary 4**

*If* *K is a PF*$\mathcal {G^*}$*OS in PF*$\mathfrak {F_{p}^*}$*FDS and PF*$\mathcal {P^*}$*S* $(\mathfrak {X}, \mathfrak {F_{p}^*})$,* then* *K* *is a PFcs-DS in* $(\mathfrak {X}, \mathfrak {F_{p}^*})$.

### *Proof*

Let *K* is a PF$\mathcal {G^*}$OS in PF$\mathfrak {F_{p}^*}$FDS $(\mathfrak {X}, \mathfrak {F_{p}^*})$. Then $K^c$ is a PF$\mathcal {G^*}$CS in $(\mathfrak {X}, \mathfrak {F_{p}^*})$. Since $(\mathfrak {X}, \mathfrak {F_{p}^*})$ is a PF$\mathfrak {F_{p}^*}$FDS by Proposition 7.4. $K^c$ is PFsWDS in $(\mathfrak {X}, \mathfrak {F_{p}^*})$ and thus *K* is a PFcs-DS in $(\mathfrak {X}, \mathfrak {F_{p}^*})$. $\square$

### **Proposition 34**

*If* *F* *is a PF*$\mathcal {G^*}$*OS in PF*$\mathfrak {F_{p}^*}$*FDS and PF*$\mathcal {P^*}$*S* $(\mathfrak {X}, \mathfrak {F_{p}^*})$, *then there exists a PF*$\mathfrak {F_{p}^*}$*RCS* *N in* $(\mathfrak {X}, \mathfrak {F_{p}^*})$ *such that* $N \subseteq cl_{\mathfrak {F_{p}^*}}(F)$.

### *Proof*

Let *F* is a PF$\mathcal {G^*}$OS in $(\mathfrak {X}, \mathfrak {F_{p}^*})$. Since $(\mathfrak {X}, \mathfrak {F_{p}^*})$ is PF$\mathfrak {F_{p}^*}$FDS and PF$\mathcal {P^*}$S by Proposition 7.4. *F* is PFsWDS in $(\mathfrak {X}, \mathfrak {F_{p}^*})$. By Proposition 4.13. there exists a PF$\mathfrak {F_{p}^*}$RCS *N* in $(\mathfrak {X}, \mathfrak {F_{p}^*})$ such that $N \subseteq cl_{\mathfrak {F_{p}^*}}(F)$. $\square$

### **Corollary 5**

*If* *K is a PF*$\mathcal {G^*}$*OS in PF*$\mathfrak {F_{p}^*}$*FDS and PF*$\mathcal {P^*}$*S* $(\mathfrak {X}, \mathfrak {F_{p}^*})$, *then there exists a PF*$\mathfrak {F_{p}^*}$*ROS* *T* *in* $(\mathfrak {X}, \mathfrak {F_{p}^*})$ *such that *$int_{\mathfrak {F_{p}^*}}(K)\subseteq T$.

### *Proof*

Let *K* be a PF$\mathcal {G^*}$OS in $(\mathfrak {X}, \mathfrak {F_{p}^*})$. Then $K^c$ is a PF$\mathcal {G^*}$CS in $(\mathfrak {X}, \mathfrak {F_{p}^*})$. Since $(\mathfrak {X}, \mathfrak {F_{p}^*})$ is PF$\mathfrak {F_{p}^*}$FDS and PF$\mathcal {P^*}$S by Proposition 7.6., there exists a PF$\mathfrak {F_{p}^*}$RCS *N* in $(\mathfrak {X}, \mathfrak {F_{p}^*})$ such that $N \subseteq cl_{\mathfrak {F_{p}^*}}(K^c).$. This implies $N \subseteq [int_{\mathfrak {F_{p}^*}}(K)]^c$ and $int_{\mathfrak {F_{p}^*}}(K)\subseteq N^c$. Let $T= N^c$. Then *T* is a PF$\mathfrak {F_{p}^*}$ROS in $(\mathfrak {X}, \mathfrak {F_{p}^*})$ and $int_{\mathfrak {F_{p}^*}}(K)\subseteq T$. $\square$

### **Proposition 35**

*If* *K is a PF*$\mathcal {G^*}$*OS in a PF*$\mathfrak {F_{p}^*}$*FDS and PF*$\mathcal {P}^*$*S, then*

(i) $$cl_{\mathfrak {F_{p}^*}}(int_{\mathfrak {F_{p}^*}}(K)) \ne 1_{\mathfrak {X}}$$ in $$(\mathfrak {X}, \mathfrak {F_{p}^*})$$.
(ii) *K* is not a PFDS in $(\mathfrak {X}, \mathfrak {F_{p}^*})$.

### *Proof*

(*i*) Let *K* be a PF$\mathcal {G^*}$OS in $(\mathfrak {X}, \mathfrak {F_{p}^*})$. Then $K^c$ is a PF$\mathcal {G^*}$CS in $(\mathfrak {X}, \mathfrak {F_{p}^*})$. Since $(\mathfrak {X}, \mathfrak {F_{p}^*})$ is PF$\mathfrak {F_{p}^*}$FDS and PF$\mathcal {P^*}$S, by Proposition 7.4 $K^c$ is a PFsWDS and $int_{\mathfrak {F_{p}^*}}(cl_{\mathfrak {F_{p}^*}}(K^c)) \ne 0_{\mathfrak {X}}$ in $(\mathfrak {X}, \mathfrak {F_{p}^*})$. Then $[cl_{\mathfrak {F_{p}^*}}int_{\mathfrak {F_{p}^*}}(K)]^c \ne 0_{\mathfrak {X}}$ and $cl_{\mathfrak {F_{p}^*}}(int_{\mathfrak {F_{p}^*}}(K)) \ne 1_{\mathfrak {X}}$ in $(\mathfrak {X}, \mathfrak {F_{p}^*})$.

(*ii*) Since $(\mathfrak {X}, \mathfrak {F_{p}^*})$ is a PF$\mathcal {P^*}$S, the PF$\mathcal {G^*}$OS *K* is a PF$\mathfrak {F_{p}^*}$OS in $(\mathfrak {X}, \mathfrak {F_{p}^*})$. Also since $(\mathfrak {X}, \mathfrak {F_{p}^*})$ is a PF$\mathfrak {F_{p}^*}$FDS by Proposition 6.6, there exists a PF$\mathcal {G^*}$CS *N* in $(\mathfrak {X}, \mathfrak {F_{p}^*})$ such that $K \subseteq cl_{\mathfrak {F_{p}^*}}(N)$ and $cl_{\mathfrak {F_{p}^*}}(N)$ is PF$\mathfrak {F_{p}^*}$CS in $(\mathfrak {X}, \mathfrak {F_{p}^*})$, implies that $cl_{\mathfrak {F_{p}^*}}(K) \ne 1_{\mathfrak {X}}$ in $(\mathfrak {X}, \mathfrak {F_{p}^*})$. Hence *K* is not a PFDS in $(\mathfrak {X}, \mathfrak {F_{p}^*})$. $\square$

### **Proposition 36**

*If* *K* *is a PF*$\mathfrak {F_{p}^*}$*OS in a PF*$\mathfrak {F_{p}^*}$*FDS and PF*$\mathcal {P^*}$*S* $(\mathfrak {X}, \mathfrak {F_{p}^*})$, *then *$cl_{\mathfrak {F_{p}^*}}(K)$ *is a PF*$\mathcal {G^*}$*CS in* $(\mathfrak {X}, \mathfrak {F_{p}^*})$.

### *Proof*

Let *K* be a PF$\mathfrak {F_{p}^*}$OS in $(\mathfrak {X}, \mathfrak {F_{p}^*})$. Since $(\mathfrak {X}, \mathfrak {F_{p}^*})$ is a PF$\mathfrak {F_{p}^*}$FDS $cl_{\mathfrak {F_{p}^*}}(K)= cl_{\mathfrak {F_{p}^*}}(F)$, where *F* is PF$\mathcal {G^*}$CS in $(\mathfrak {X}, \mathfrak {F_{p}^*})$. Also $(\mathfrak {X}, \mathfrak {F_{p}^*})$ is a PF$\mathcal {P^*}$S, the PF$\mathcal {G^*}$CS *F* is a PF$\mathfrak {F_{p}^*}$CS in $(\mathfrak {X}, \mathfrak {F_{p}^*})$ and then $cl_{\mathfrak {F_{p}^*}}(F)=F$. Then $cl_{\mathfrak {F_{p}^*}}(K)=F$, where *F* is a PF$\mathcal {G^*}$CS in $(\mathfrak {X}, \mathfrak {F_{p}^*})$. Hence $cl_{\mathfrak {F_{p}^*}}(K)$ is a PF$\mathcal {G^*}$CS in $(\mathfrak {X}, \mathfrak {F_{p}^*})$. $\square$

### **Corollary 6**

*If* *F* *is a PF*$\mathfrak {F_{p}^*}$*CS in PF*$\mathfrak {F_{p}^*}$*FDS and PF*$\mathcal {P^*}$*S* $(\mathfrak {X}, \mathfrak {F_{p}^*})$, *then* $int_{\mathfrak {F_{p}^*}}(F)$ *is a PF*$\mathcal {G^*}$*OS in *$(\mathfrak {X}, \mathfrak {F_{p}^*})$.

### *Proof*

Let *F* be PF$\mathfrak {F_{p}^*}$CS in $(\mathfrak {X}, \mathfrak {F_{p}^*})$. Then $F^c$ is a PF$\mathfrak {F_{p}^*}$OS in PF$\mathfrak {F_{p}^*}$FDS and PF$\mathcal {P^*}$S $(\mathfrak {X}, \mathfrak {F_{p}^*})$, by Proposition 7.9. $cl_{\mathfrak {F_{p}^*}}(F^c)$ is PF$\mathcal {G^*}$CS in $(\mathfrak {X}, \mathfrak {F_{p}^*})$. By Proposition 4.11. $cl_{\mathfrak {F_{p}^*}}(F^c)=[int_{\mathfrak {F_{p}^*}}(F)]^c$. Hence $int_{\mathfrak {F_{p}^*}}(F)$ is a PF$\mathcal {G^*}$OS in $(\mathfrak {X}, \mathfrak {F_{p}^*})$. $\square$

## Comparison with existing theory

In the existing literature, the concept of Pythagorean fuzzy frames has been primarily studied as an extension of classical fuzzy and intuitionistic fuzzy frameworks, focusing mainly on the representation of uncertainty and partial truth in algebraic or decision-theoretic contexts. Earlier studies have concentrated on defining Pythagorean fuzzy sets, relations and topological spaces establishing their basic properties and operations. However, the structural development of Pythagorean fuzzy frames particularly from a topological viewpoint has remained limited.

The current study advances this theoretical foundation by introducing a Pythagorean fuzzy $\mathfrak {F_{p}^*}$ structure space which generalizes the notion of open sets in topology using frame theory. Unlike previous works that treated fuzzy frames as abstract algebraic constructs this research systematically defines and investigates new topological components within the Pythagorean fuzzy frame setting such as Pythagorean fuzzy $\mathcal {G^*}$ closed sets, dense, nowhere dense and somewhere dense sets, $\mathfrak {F_{p}^*}$ continuous functions and separation axioms.

Furthermore, the study introduces and explores new spaces Pythagorean fuzzy $\mathfrak {F_{p}^*}$ fraction dense spaces and Pythagorean fuzzy $\mathcal{P^*}$spaces which have not been previously formulated in existing theories. These additions enrich the structural and functional understanding of Pythagorean fuzzy frames bridging the gap between abstract lattice theoretic foundations and topological generalizations.

In summary, while existing theories laid the groundwork for representing uncertainty using Pythagorean fuzzy sets and relations, the current study extends this framework by developing a comprehensive topological model based on frames offering a more generalized and flexible approach for analyzing fuzzy structures.

## Conclusion and future work

In this study, Pythagorean fuzzy frames, Pythagorean fuzzy $\mathfrak {F_{p}^*}$ structure space, Pythagorean fuzzy $\mathfrak {F_{p}^*}$ continuous function, separation axioms on Pythagorean fuzzy $\mathfrak {F_{p}^*}$ structure space is established. Various sets are investigated in Pythagorean fuzzy $\mathfrak {F_{p}^*}$ structure space. Pythagorean fuzzy $\mathfrak {F_{p}^*}$ fraction dense space is introduced to study the defined sets in structure space. Several characterizations of PF$\mathfrak {F_{p}^*}$FDS are investigated. Also the relationship between PF$\mathfrak {F_{p}^*}$ CS, PF$\mathfrak {F_{p}^*}$OS, PF$\mathcal {G^*}$, PF$\mathcal {G^*}$CS are are explored in PF$\mathfrak {F_{p}^*}$FDS and PF$\mathcal {P^*}$S. In future, relationship between PF$\mathfrak {F_{p}^*}$FDS and various other spaces like baire space, sober space, cellular spaces can be investigated. Pythagorean fuzzy coframes, Pythagorean fuzzy subframes can be discussed in various Pythagorean fuzzy topological spaces.

The system of open sets of a space where the relationships between these sets meet certain lattice qualities is represented by an algebraic structure called a frame in topology. Each open set can be described in terms of degrees of membership and non-membership expressing uncertainty and partial truth conditions that are prevalent in many real-world systems by extending this concept into a Pythagorean fuzzy frame. For instance, a medical diagnosis system that groups patients according to test results and symptoms. A symptom may be categorized as present or absence using traditional crisp sets. In reality though, a lot of symptoms are not absolute; a patient may display them to some degree. Each symptom (like an open set) can be represented with Pythagorean fuzzy membership values using Pythagorean fuzzy frames which capture the degree to which a symptom confirms or refutes a diagnosis. The Pythagorean fuzzy component captures the uncertainty present in medical assessment while the frame structure guarantees the algebraic preservation of links among various symptoms (intersections, unions, and inclusion relations). This makes decision-making more precise and adaptable particularly in cases where clinical data are inconsistent or lacking. Similarly, this approach can be extended to: Risk analysis, where uncertain parameters influence investment or safety decisions. Artificial intelligence, where fuzzy frames support learning and reasoning under uncertainty. Engineering control systems, where approximate measurements require adaptive modeling.

## Data Availability

No datasets were generated or analysed during the current study.
